# Comprehensive Analysis of Pyrazoline Analogs: Exploring Their Antioxidant Potential as Biofuel Additives

**DOI:** 10.1021/acsomega.5c00398

**Published:** 2025-07-31

**Authors:** Patricia R. S. Wenceslau, Antônio S. N. Aguiar, Vitor S. Duarte, Leonardo R. de Almeida, Chris H. J. Franco, Gilberto L. B. de Aquino, Jaqueline E. de Queiroz, Adriano O. Maldaner, Hamilton B. Napolitano

**Affiliations:** † Grupo de Química Teórica e Estrutural de Anápolis, 271384 Universidade Estadual de Goiás, Anápolis 75132-400, Goiás, Brazil; ‡ Laboratório de Novos Materiais, Universidade Evange’lica de Goiás, Anápolis 75132-400, Goiás, Brazil; § MINDlab: Molecular Design & Innovation Laboratory, Centro de Química Estrutural, Institute of Molecular Sciences, Instituto Superior Técnico, 72971 Universidade de Lisboa, Lisboa 1049-001, Portugal; ∥ Instituto Nacional de Criminalística, Diretoria Técnico Cientifica, 383815 Polícia Federal, Federal District, Brasília 70037-900, Brazil

## Abstract

Global dependence on fossil fuels raises environmental and energy concerns, encouraging the search for renewable sources, such as biodiesel. However, their limited oxidative stability compromises their commercial viability. This study investigates two pyrazoline analogues (CNF and CNO) as potential antioxidant additives for biodiesel using a combined molecular modeling and machine learning approach. Supramolecular analysis revealed that C–H···O and C–H···π interactions influence molecular stability. Chemical reactivity descriptors indicated that the methoxy group (−OCH_3_) in CNO enhances the electron-withdrawing effect, making it more susceptible to oxidation. Kinetic evaluation predicted that both compounds have intermediate oxidation rates when exposed to hydroxyl radicals, with a performance comparable to widely used commercial antioxidants. These results suggest that pyrazoline analogues are promising candidates to mitigate the oxidative degradation of biodiesel, contributing to the greater efficiency and sustainability of biofuels.

## Graphical abstract

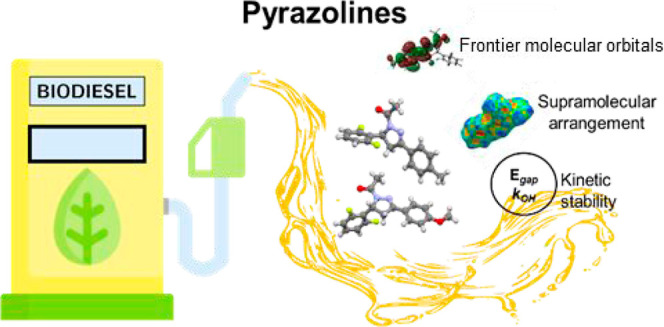

## 1 Introduction

Energy systems are fundamental to modern civilization, driving technological progress and improving the quality of life. Currently, global energy demands are primally satisfied by fossil fuels, such as petroleum-based sources.^1,2^ However, these resources are nonrenewable and contribute significantly to environmental degradation. The combustion of fossil fuels accounts for approximately 82% of greenhouse gas (GH) emissions, primarily through carbon dioxide (CO_2_) release.^3^ Additionally, the extensive use of these fuels in engines contributes to environmental pollution by releasing unburned hydrocarbons, carbon monoxide (CO), and nitrogen oxides (NO_*x*_), resulting in significant environmental impacts, such as global warming, air pollution, and acidification.^4,5^ In response to these challenges, the United Nations Sustainable Development Goals (SDGs) emphasize the need for a global energy transition and enhancing environmental quality.^6,7^

The seventh SDG, in particular, calls for global commitments toward a substantial increase in the share of renewable energy and improvements in energy efficiency by 2030, ensuring access to affordable, reliable, sustainable, and modern energy for all.^8^ In this context, biofuels have emerged as promising alternatives due to their renewable nature and reduced lifecycle carbon emissions compared to fossil fuels.^5,9^ It can contribute by reducing greenhouse gas emissions and helping to meet international carbon reduction commitments.^10–12^ Biodiesel, derived from vegetable oils, animal fats, and used cooking oil. It is biodegradable and a sustainable, less toxic, and has low sulfur and aromatic hydrocarbons.^13^ Despite its advantages, biodiesel suffers from poor oxidative stability, especially during prolonged storage, which limits its efficiency.^10,14^ This vulnerability arises from its high content of unsaturated fatty acids, which are prone to oxidative degradation in the presence of oxygen, heat, or light.^15^

Oxidation leads to the formation of peroxides, acids, and insoluble particulates that affect the fuel performance. A promising strategy to address this issue is the incorporation of organic antioxidant additives into biofuels, which can inhibit radical formation, enhance fuel stability, and potentially reduce harmful emissions.^10,11,16^ Among various antioxidant candidates, heterocyclic compounds—particularly those with electron-rich structures—have shown promise due to their radical scavenging abilities. Pyrazolines, a class of five-membered heterocyclic compounds with two adjacent nitrogen atoms, have received attention for their chemical versatility,^17^ including antimicrobial activity^18^ and antioxidant properties.^19^ Despite the growing interest in antioxidants for biodiesel, the application of pyrazoline derivatives for this purpose remains underexplored. Given their ability to neutralize reactive oxygen species (ROS), investigating this class of compounds may open new possibilities for improving the oxidative stability of biodiesel.

Although the additives under real-world conditions are used in practically low concentrations, combustion may generate trace amounts of nitrogen and fluorine-containing emissions; it is important to note that nitrogen-containing compounds must be carefully evaluated for fuel applications, as they can influence NO_*x*_ emissions during combustion. However, the antioxidant behavior of such compounds is often effective at low concentrations, potentially minimizing their effects on combustion products. A deeper understanding of their oxidative inhibition mechanisms is essential for evaluating their practical viability and environmental safety to design new compound candidates for improving biodiesel performance.^19^

In this study, we present a theoretical investigation into the antioxidant potential of two pyrazoline-based compounds as antioxidant additives for biodiesel. Employing density functional theory (DFT), frontier molecular orbitals (FMO) analysis, Fukui functions, and machine learning simulations, we evaluate their capacity to neutralize ROS and enhance the oxidative stability of biodiesel. This work aims to provide insights from a computational foundation for the rational design of novel antioxidant additives with high efficiency and potential to enhance biodiesel’s oxidative stability and performance.

## 2 Experimental and Computational Procedures

### 2.1 Synthesis and Spectroscopic Analysis

The 1-(5-(2,6-difluorophenyl-4,5-dihydro-3-*p*-toluyllpyrazol-1-yl))­ethenone^20^ (CNF) and 1-(5-(2,6-difluorophenyl)-4,5-dihydro-3-(4-methoxyphenyl)­pyrazol-1-yl)­ethenone (CNO) were obtained by the reaction of chalcone correspondent (1 mmol) and 1.2 mmol of hydrazine 80% using acetic acid (1 mL)^20^ (Scheme 1). The reactions were undertaken on microwave-assisted heating using the reactor CEM Discover (North Caroline, USA) at 150 °C for 5 min. After the reactions were completed, the obtained precipitates were collected by vacuum filtration and purified by crystallization in absolute ethanol. The progress of the reactions was monitored by using silica gel plates (TLC 60 UV254). The use of microwaves allowed a high yield of the product in a short reaction time. The pyrazoline analogs were then characterized by ^1^H and ^13^C nuclear magnetic resonance (NMR) spectroscopy (Figures S1–S4). The white crystalline solids of CNF (yield 78%) and CNO (yield 70%) were obtained by direct evaporation.

**Scheme 1:**
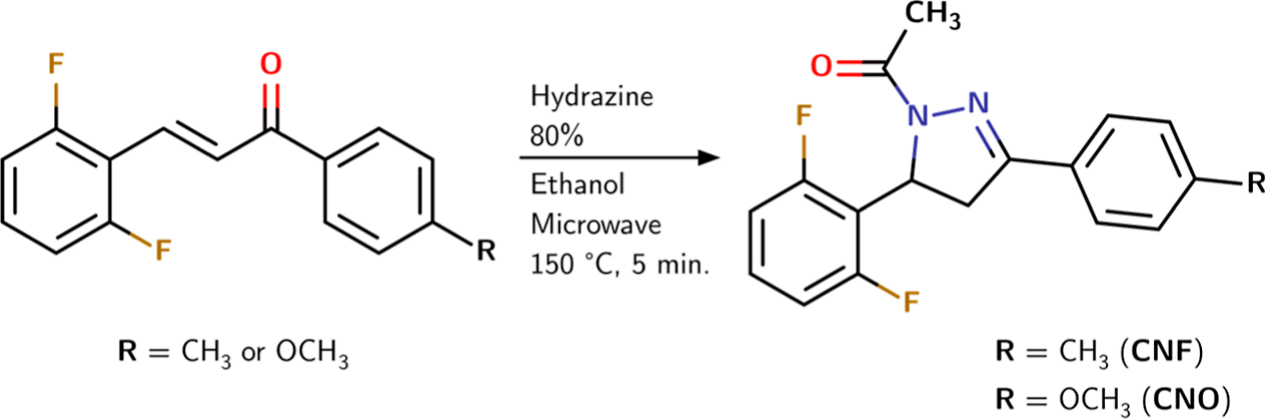
Synthesis of the Pyrazole Derivatives CNO and CNF

#### 2.1.1 Synthesis of CNF

1 mmol of (*E*)-3-(2,6-difluorophenyl)-1-*p*-tolyl-prop-2-en-1-one (C_16_H_14_F_2_O, 258.26 g·mol^–1^, 0.2585 g) and 1 mmol of hydrazine (C_2_H_4_N_2_, 32.05 g·mol^–1^; 0.0321 g). Product: C_18_H_16_F_2_N_2_O, 314.33 g·mol^–1^; white solid; melting point of 112.2 °C; 78% yield. NMR spectroscopy (CNF): ^1^H (500 MHz, acetone): δ 7.72 (d, *J* = 8.2 Hz, 2H), 7.34 (ddd, *J* = 8.4 and 6.4 Hz, 1H), 7.29 (d, *J* = 8.2 Hz, 2H), 6.97 (dd, *J* = 8.4 and 8.4 Hz, 1H), 5.84 (dd, *J* = 12.7, 6.3 Hz, 1H), 3.89 (dd, *J* = 17.9, 12.7 Hz, 1H), 3.32 (dd, *J* = 17.9, 6.3 Hz, 1H), 2.38 (3, 2H), 2.24 (s, 3H). ^13^C (126 MHz, acetone) δ: 167.38 (s), 160.93 (dd, *J* = 248.3, 8.0 Hz), 153.58, 140.25, 129.51 (t, *J* = 10.8 Hz), 129.30, 128.96, 126.50, 111.48 (d, *J* = 25.6 Hz), 49.90, 39.74, 28.95 (dt, *J* = 38.8, 19.4 Hz), 20.85 (s, *J* = 41.3 Hz), 20.52.

#### 2.1.2 Synthesis of CNO

1 mmol of (*E*)-3-(2,6-difluorophenyl)-1-*p*-methoxy-prop-2-en-1-one (C_16_H_14_F_2_O, 274,26 g·mol^–1^, 0.2745 g) and 1 mmol of hydrazine (C_2_H_4_N_2_, 32.05 g·mol^–1^; 0.0321 g). Product: C_18_H_16_F_2_N_2_O_2_, 314.12 g·mol^–1^; 70% yield; white solid; IR (KBr) ν/cm^–1^ 2936; 2840; 1625; 1471; 1255; 834; 784; 730; 700. GC/MS retention time of 7.2 min; *m*/*z* 330.08; 288.08; 175.06; 134.03 and 43.03. NMR spectroscopy (CNO): ^1^H NMR (600 MHz, CDCl_3_, δ) 7.80–7.62 (m, 2H), 7.23 (tt, *J* = 8.3, 6.3 Hz, 1H), 7.01–6.92 (m, 2H), 6.88 (t, *J* = 8.6 Hz, 2H), 5.87 (dd, *J* = 12.6, 6.2 Hz, 1H), 3.88 (s, 3H), 3.72 (dd, *J* = 17.5, 12.6 Hz, 1H), 3.28 (dd, *J* = 17.5, 6.2 Hz, 1H), 2.37 (s, 3H). ^13^C (151 MHz, CDCl_3_, δ) 168.62, 161.29, 160.08 (dd, 250 and 8 Hz), 153.26, 129.27 (t, 10 Hz), 128.11, 124.04, 114.16, 111.61 (d, 25.4 Hz), 55.42, 49.93, 40.07, 21.80.

### 2.2 Solid State Analysis

The single crystal X-ray diffraction data for the CNF were obtained on a SuperNova diffractometer (AtlasS2 detector type) with Cu Kα (λ = 1.54184 Å) at room temperature (≈292.8 K). The crystal of CNO was collected at the MANACA beamline/facility of SIRIUS, the Brazilian Synchrotron Light Laboratory (LNLS), under cryogenic temperature (≈120 K), using parathone as a cryoprotectant and the wavelength of (λ = 0.67019 Å). Data were fully processed using the MNCAutoproc pipeline with XDS.^21^ Both crystal structures were solved using SHELXS^22^ and refined SHELXL,^23^ which can be found on the platform OLEX2.^24^ All graphics and figures and the supramolecular interactions are generated by Mercury.^25^ The crystallographic data of both molecules can be found in the Cambridge Crystallographic Data Centre (CCDC),^26^ through the codes: 2393018 (CNF) and 2393138 (CNO).

The Hirshfeld surface (HS) is a tool used to analyze intermolecular interactions in crystals through electron density analysis. These surfaces are based on electron density and indicate regions of possible interactions, in which red dots indicate regions with strong intermolecular contacts, while blue and white regions indicate weak or absent contacts. The three-dimensional graphics are calculated using the distances between the internal atom adjacent to the surface (*d*_i_) and the closest external atom (*d*_e_). The combination of *d*_i_ and *d*_e_ generates *Fingerprints*, which are 2D graphics that visually represent intermolecular interactions. These *Fingerprints* are unique to each crystal structure and enable the quantitative analysis of various types of intermolecular contacts.^27^

### 2.3 Molecular Modeling Analysis

The analysis of the molecular and electronic structures of the compounds CNF and CNO was carried out by DFT,^28,29^ implemented in the Gaussian16 software package.^30^ Theoretical calculations were conducted in the gas phase using the hybrid functional M06–2X,^31^ accompanied by the Pople basis set 6–311++G­(d,p).^32^ This level of theory is known for its satisfactory results, considering electronic correlation and being particularly suitable for noncovalent interactions.^31,33^ The inputs were constructed by using crystallographic atomic coordinates. From the energy values of the FMO,^34^ the highest occupied molecular orbital (HOMO), and the lowest unoccupied molecular orbital (LUMO), it was possible to obtain information about the chemical reactivity of the compounds, namely, the chemical potential^35^

1 $$\mu ={\left(\frac{\partial E}{\partial N}\right)}_{\upsilon }=-\frac{I+A}{2}=-\chi$$

related to charge transfer from a species with higher chemical potential, μ_large_, to one with lower chemical potential, μ_small_, the chemical hardness^35,36^

2 $$\eta =\frac{1}{2}{\left(\frac{\partial^{2}E}{\partial N^{2}}\right)}_{\upsilon }=\frac{I-A}{2}$$

measuring resistance to deformation of the electron cloud during chemical processes, and the global electrophilicity index^37^

3 $$\omega =\frac{\mu^{2}}{2\eta }$$

measuring energy stabilization when the system acquires electronic charge from the environment. In eqs 1 and 2, *E* is the energy of the system, *N* is the number of particles, υ is the external potential, χ is the electronegativity, *I* ≅ −*E*_HOMO_ is the ionization potential, and *A* ≅ −*E*_LUMO_ is the electron affinity. The molecular electrostatic potential (MEP) map was used to identify the nucleophilic and electrophilic regions of the molecules,^38,39^ and the electrostatic potentials *V*(**r**) values at the **r** point^40^ is defined as

4 $$V\left(r\right)=\sum_{\alpha }\frac{Z_{\alpha }}{\left|r_{\alpha }-r\right|}-\int \frac{\rho \left(r^{\prime }\right)}{\left|r^{\prime }-r\right|}dr^{\prime }$$

where *Z*_α_ is the charge of nuclei α at point **r**_α_ and ρ­(**r**′) is the charge density at the point **r**′,^38^ From the Fukui function,^41,42^ it was possible to determine the sites where the nucleophilic, electrophilic, and radical attacks of the molecules of the compounds occur.

### 2.4 Machine Learning Procedures

The oxidation reactions promoted by free-radical compounds were simulated by *py*SiRC,^43^ a machine-learning computational platform for reaction kinetics. To mimic the oxidative environment commonly associated with biodiesel degradation, the hydroxyl radical (^•^OH)—an archetypal system of degradation reactions—was selected as a model oxidizing species due to its high reactivity and representative role in the radical–mediated degradation pathway. The referred kinetic parameters used for training this ML platform were catalogued under standard conditions, 25 °C and 1 mol·L^–1^ in the aqueous phase.^43^ Sanches-Neto and co-authors^43^ reported that data sets were randomly split into a training set (80%) and test set (20%). The model employed the XGBoost algorithm in conjunction with Morgan fingerprints as molecular descriptors. The reported performance metrics included a training set correlation coefficient (*R* ^2^, training set = 0.937), test set Pearson correlation coefficient of prediction (*r* ^2^ = 0.840), root-mean-square error (RMSE, training set = 0.101; RMSE_ext_, test set = 0.085), and test set external validation coefficient (*Q*_ext_^2^ = 0.707).

In the present work, we adopted the framework using the Morgan fingerprint as a structural descriptor and the XGBoost algorithm to predict the reaction rate constants (*k*_OH_) for the oxidative attack promoted by hydroxyl radical to the compounds in the B20 blends.^44^ The calculations included the major constituents of diesel (represented by C_10_H_20_ molecule), biodiesel (BD)—represented by its key fatty acid methyl esters (FAMEs): methyl 9-octadecenoate (M9OD, C_19_H_38_O_2_—19.98%), methyl palmitate (MPAL, C_17_H_34_O_2_—12.87%), and methyl 8,11-octadecenoate (M8OD, C_19_H_34_O_2_—10.22%).^45^ In addition to the baseline components, the predicted rate constants were also calculated for the two pyrazoline-based molecules under study: CNF (C_18_H_16_F_2_N_2_O) and CNO (C_18_H_16_F_2_N_2_O_2_). For comparative purposes, the performance of these compounds was benchmarked against that of a series of established antioxidant additives. These included previously reported molecules from our group, Chal01, Chal05,^46^ and TMC20,^47^ and well-known commercial antioxidants cited in the literature:^48^ butylated hydroxytoluene (BHT), *tert*-butyl hydroquinone (TBHQ), butylated hydroxyanisole (BHA), propyl gallate (PG), pyrogallol (PY), and gallic acid (GA).

## 3 Results and Discussion

### 3.1 Solid State Characterization

The CNF and CNO molecules crystallized in the *C*2/*c* space group. Table 1 summarizes the refinement parameters of the molecules, and the ORTEP representation is presented in Figure 1. Further crystallographic parameters are presented in Tables S1–S10. The CNF and CNO compounds have the C7 chiral center in a centrosymmetric space group (*C*2/*c*), and the S configuration was chosen for discussion. The structures of the CNF and CNO molecules exhibit notable similarities, including the presence of two fluorine atoms in ortho positions on aromatic ring 1, and a pyrazole group on ring 2. The primary difference between them lies in aromatic ring 3. In the CNF molecule, carbon C_15_ (para position) is bonded to a methyl group (−CH_3_), whereas in the CNO molecule, the same carbon (para position) is bonded to an oxygen atom, forming a methoxy group (O–CH_3_). The overlap between the CNF and CNO structures reveals a deviation in aromatic ring 3, with a difference of 9.14° in this region. Additionally, the substitution region from a methyl group (CNF) to a methoxy group (CNO) shows a variation of 4.04° (Figure 1). The largest deviation was observed in the region binding to the nitrogen atom (N_2_), with a difference of 15.60°. These discrepancies in molecular conformation could affect the physicochemical properties of each molecule.

**Table 1:** Crystal Data and Structure Refinement Information of the Pyrazoline Derivatives CNF and CNO

compound	CNF	CNO
chemical formula	C_18_H_16_F_2_N_2_O	C_18_H_16_F_2_N_2_O_2_
temperature	292.80(5) K	120 K
wavelength	(λ = 1.54184) Å	(λ = 0.67019) Å
crystal system, space group	monoclinic, *C*2/*c*	monoclinic, *C*2/*c*
unit cell dimensions	*a* = 17.6194(2) Å	*a* = 15.867(9) Å
	*b* = 7.46980(10) Å	*b* = 7.224(4) Å
	*c* = 24.2201(3) Å	*c* = 27.760(6) Å
	α = 90.000°	α = 90.000°
	β = 99.980(10)°	β = 90.131(13)°
	γ = 90.000°	γ = 90.000°
volume	3139.45(7) Å^3^	3182(3) Å^3^
*Z*, *Z*′	8, 1	8, 1
density (calculated)	1.330 g·cm^–3^	1.379 g·cm^–3^
absorption coefficient	0.834 mm^–1^	0.094 mm^–1^
*F*(000)	1312.0	1376.0
theta range for data collection	7.412–138.222	2.766–60.104
completeness to theta = 25.242°	100.0%	100.0%
refinement method	full-matrix least-squares on *F* ^2^	full-matrix least-squares on *F* ^2^
data/restraints/parameters	2914/0/211	4624/0/260
goodness-of-fit (*S*)	1.035	1.066
final *R* indices [*I* > 2σ(*I*)]	*R*_1_ = 0.0388, *wR*_2_ = 0.1068	*R*_1_ = 0.0592, *wR*_2_ = 0.1522
*R* indices (all data)	*R*_1_ = 0.0396, *wR*_2_ = 0.1075	*R*_1_ = 0.0600, *wR*_2_ = 0.1533

**Figure 1:**
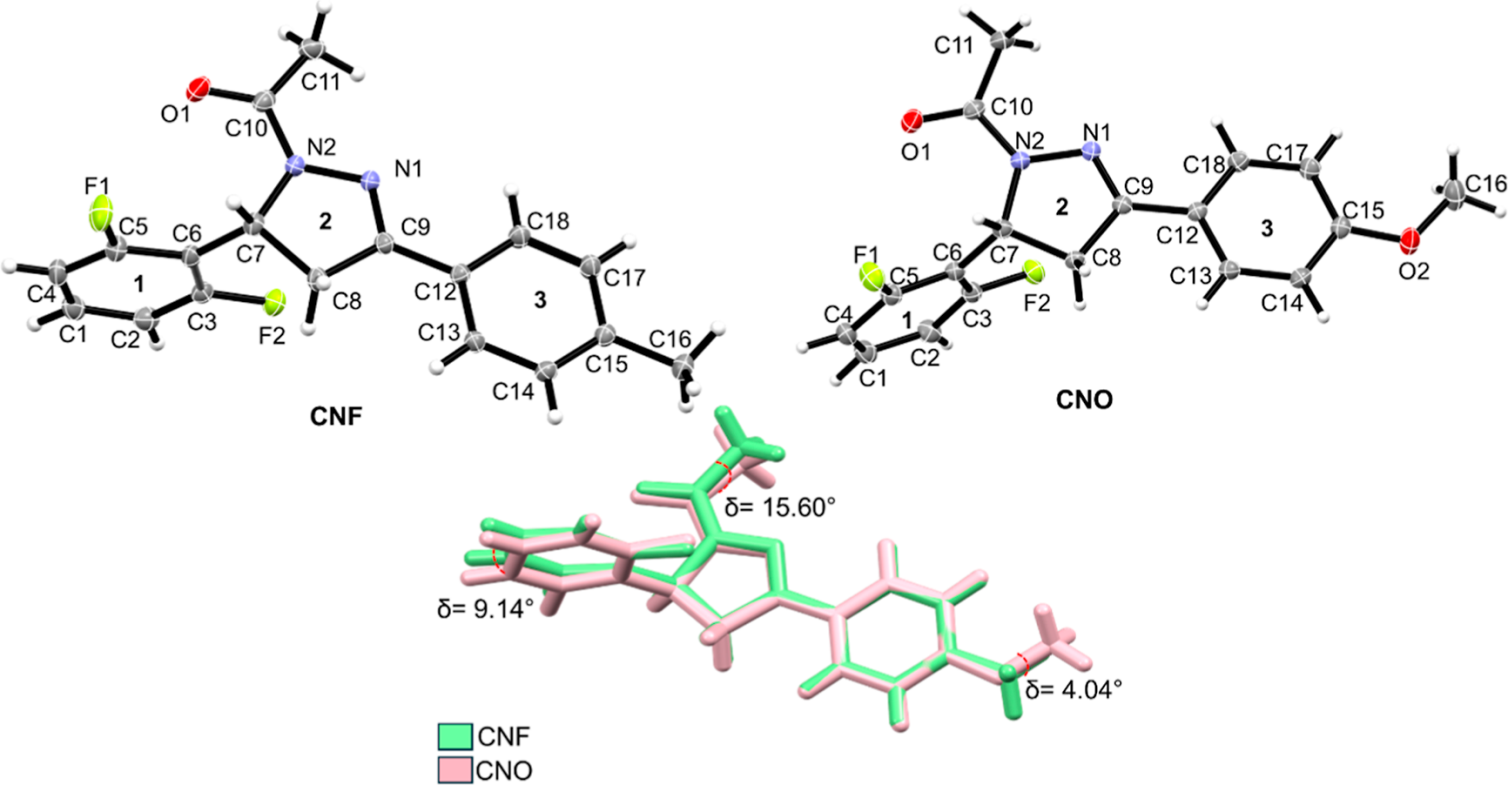
Molecular structure of the pyrazoline derivatives (CNF) and (CNO). The ORTEP type diagram of the asymmetric unit with 50% probability ellipsoids and 30% probability, respectively, showing the atomic numbering scheme CNF and CNO. Hydrogen atoms are shown as spheres of arbitrary radii. Overlap of the compounds: CNF and CNO. The molecules are identified according to their color scheme, CNF (green) and CNO (pink).

These conformational differences observed in the solid state prompted further investigation through computational analyses. Relaxed scan calculations indicate that the rotatable bonds (C_9_–C_12_, C_6_–C_7_, and N_2_–C_10_) in both molecules adopt conformations corresponding to their lowest energy states. In these conformations, benzene ring 3 is nearly coplanar with the pyrazole ring, forming dihedral angles of 2.02° in CNF and 4.82° in CNO. Conversely, ring 1 is almost perpendicular to the pyrazole ring with interplanar angles of 88.17° in CNF and 89.31° in CNO.

In the CNF and CNO structures, HS analysis confirmed the presence of C–H···O, C–H···F and C–H···π interactions. These interactions likely play a crucial role in orienting the molecules within the crystal and contribute to the stabilization of the overall structure. These interactions are crucial for maintaining molecular stability within the crystal. The main interactions of the supramolecular arrangement for CNF and CNO are presented in Table 2. The differences in crystal packing observed between the CNF and CNO molecules may be related to variations in their molecular interactions. The molecular packing for CNF and CNO can be observed along the *a*, *b*, and *c* axes in (Figure S5). CNO exhibits a more open packing arrangement compared with CNF, which adopts a tightly interlocking pattern. This variation in packing may result in differences in molecular properties, such as density and solubility.

**Table 2:** Geometries of the Intermolecular Interactions, Distances Are in Angstrom (Å), and Angles Are in Degree (°) for CNF and CNO

molecule	D–H···A	*d*(D–H) (Å)	*d*(H···A) (Å)	*d*(D···A) (Å)	*d*(D–H···A) (°)	symmetry code
CNF	C_16_–H_16b_···F_1_	0.960	2.662	3.536	151.72	*x*, 1 – *y*, −1/2 + *z*
	C_18_–H_18_···F_2_	0.930	2.573	3.440	155.16	1.5 – *x*, 1.5 – *y*, 1 – z
	C_1_–H_1_···O_1_	0.930	2.578	3.213	125.92	1.5 – *x*, −1/2 + *y*, 1/2 – *z*
	C_2_–H_2_···O_1_	0.930	2.445	3.359	167.27	*x*, 1 + *y*, *z*
	C_8_–H_8a_···Cg_1_	0.970	2.702	3.566	148.67	1–*x*, 1 – *y*, 1 – *z*
CNO	C_18_–H_18_···F_2_	0.963	2.527	3.268	133.77	1/2 – *x*, 1.5 – *y*, −*z*
	C_1_–H_1_···O_1_	0.930	2.697	3.309	122.11	1/2 – *x*, 1/2 + *y*, 1/2 – *z*
	C_2_–H_2_···O_1_	0.930	2.364	3.257	161.10	*x*, 1 + *y*, *z*
	C_11_–H_11b_···Cg_1_	0.960	2.975	3.727	136.17	1/2 – *x*, 1.5 – *y*, −*z*

The C–H···F (Figure 2a,b) and C–H···O (Figure 3a,b) interactions in both CNF and CNO molecules are confirmed by the donor and acceptor regions identified in the HS analysis (Figures 2c,d, and 3c,d). The CNF compound exhibits two C–H···F interactions: C_16_–H_16b_···F_1_ [*d*(D···A) = 3.536 Å] and C_18_–H_18_···F_2_ [*d*(D···A) = 3.440 Å], whereas the CNO compound displays only one, involving equivalent atoms as the latter—C_18_–H_18_···F_2_—but with a shorter donor–acceptor distance of 3.268 Å. Notably, the C_2_–H_2_···O_1_ interaction exhibits the shortest distance in both structures [*d*(D···A) = 3.359 Å for CNF and 3.257 Å for CNO], indicating a stronger interaction. C–H···O interactions stabilize the molecular structure by reducing flexibility and ensuring a favorable conformation for antioxidant activity. In antioxidants, these interactions enhance proton transfer and radical quenching, which are crucial for preventing oxidative damage. Compounds with strong C–H···O interactions tend to be more stable and effective at scavenging free radicals, which contribute to oxidative degradation.^49,50^ The *Fingerprint* plots further illustrate these interactions, revealing their contribution to the overall crystal stabilization. The C–H···F interactions contribute 16.2% and 16.6% (Figure 2e,f), and the C–H···O interactions represent 8.6% in CNF and 12.5% in CNO, respectively (Figure 3e,f).

**Figure 2:**
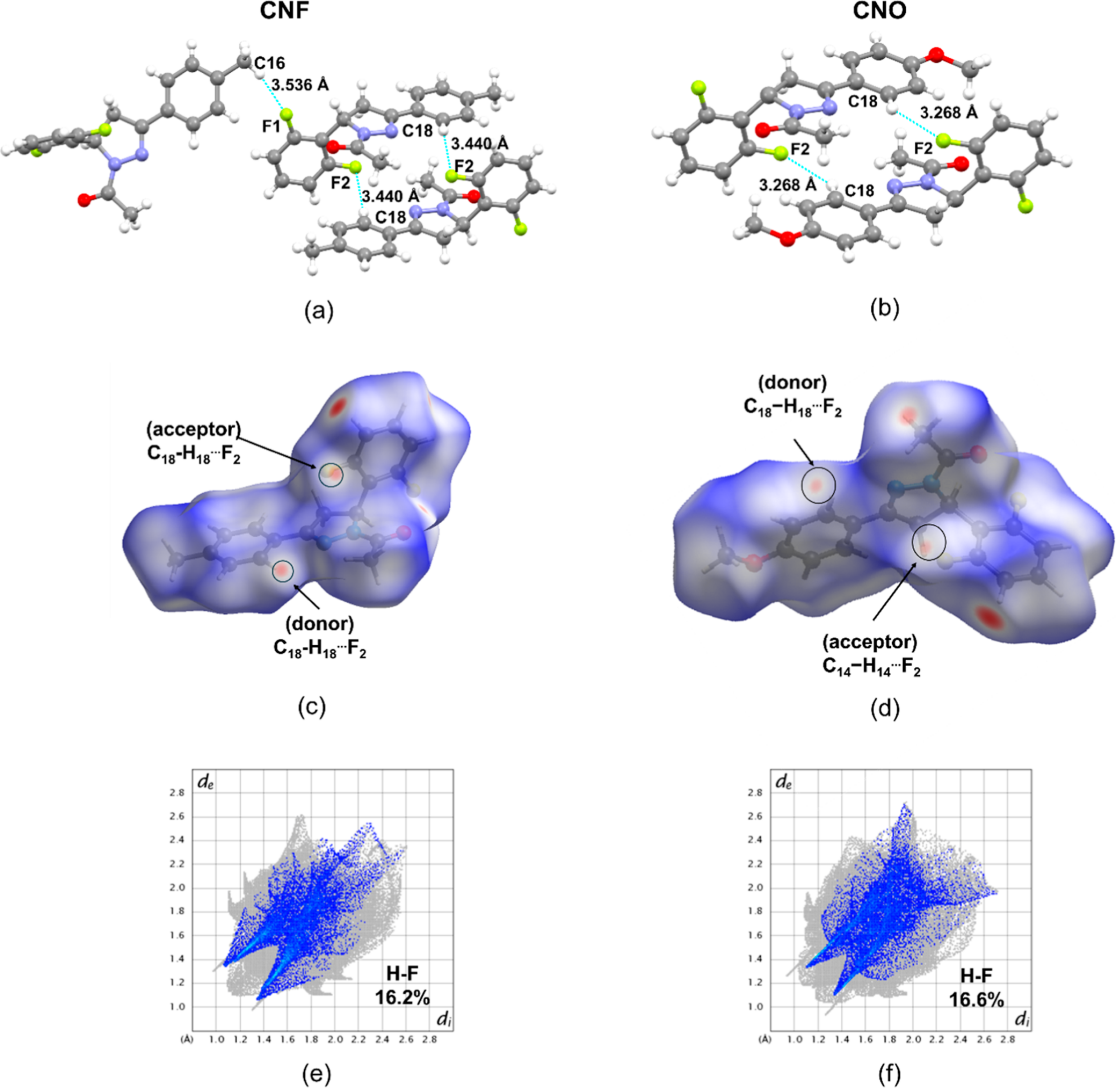
Detailed view of interactions C–H···F established of the molecule. (a) CNF and (b) CNO. The HS and the detailing view of C–H···F interactions in the supramolecular arrangement of *dnorm* surface mapped (c) CNF and (d) CNO. The 2D *Fingerprint* plot of the total contacts mapped of (e) CNF and (f) CNO.

**Figure 3:**
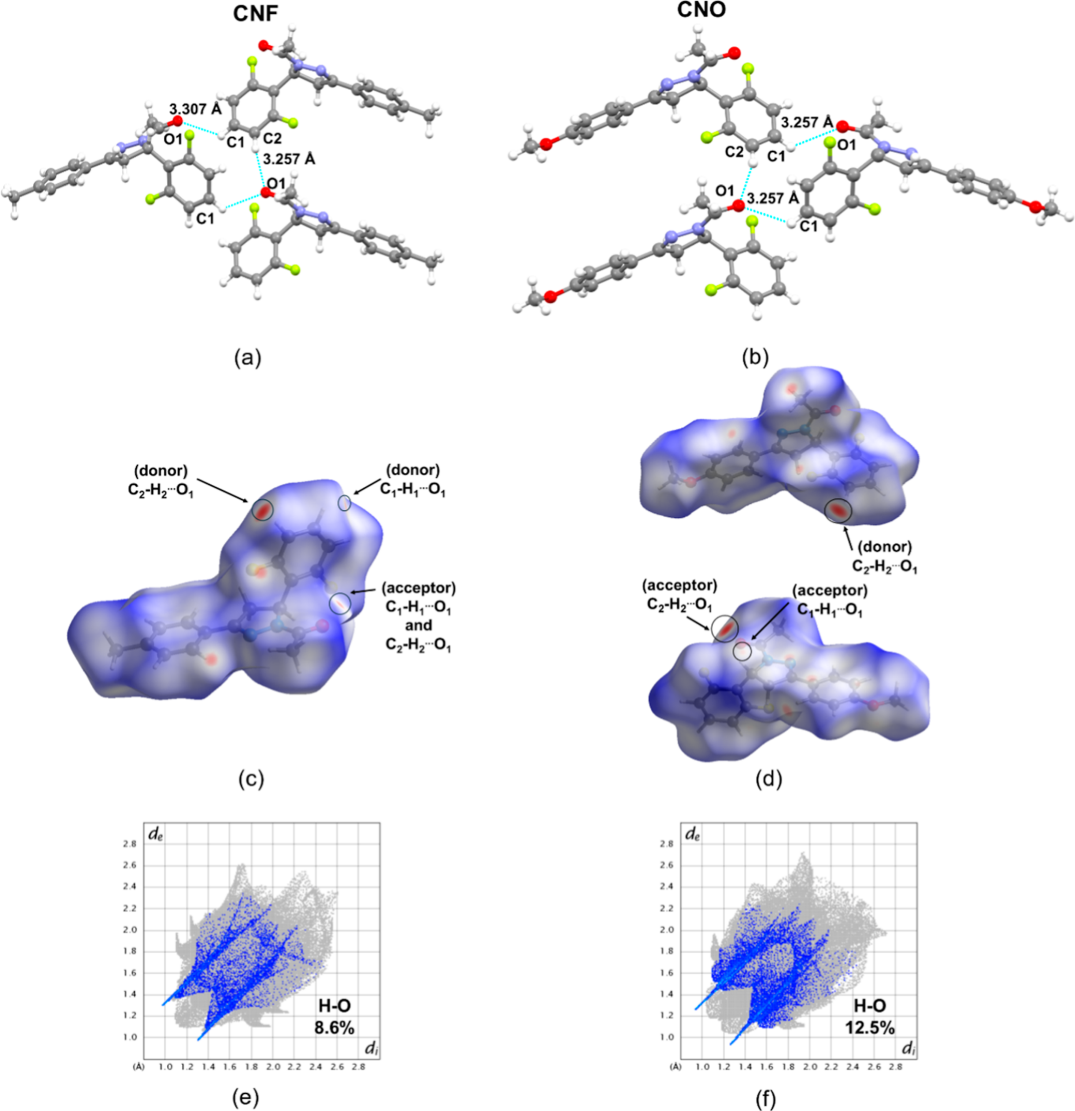
Detailed view of interactions C–H···O of the molecule. (a) CNF and (b) CNO. The HS and the detailing view of C–H···O interactions in the supramolecular arrangement of *dnorm* surface mapped (c) CNF and (d) CNO. The 2D *Fingerprint* plot of the total contacts mapped of (e) CNF and (f) CNO.

Additionally, both molecules exhibit C–H···π interactions, as identified by the HS shape index, characterized by concave red regions over the aromatic rings and convex blue regions over the C–H donor atoms. These C–H···π interactions contribute to the stabilization of molecular conformations, influencing reactivity and antioxidant efficiency by affecting electron distribution and molecular packing. Specifically, C–H···π interactions—occurring between C–H bonds and the π-electrons of aromatic rings—play a key role in stabilizing antioxidant structures by increasing molecular rigidity, thereby enhancing their interaction with radicals or ROS. These interactions also help position the antioxidant more effectively against oxidation. For antioxidants with aromatic or conjugated systems, such as the pyrazoline derivatives in our study, these interactions enhance radical scavenging by promoting charge delocalization.^51^

Figure 4a,b shows that C–H···π interactions promote dimer formation: in CNF, through C_8_–H_8a_···C_g1_ [*d*(C···C_g_) = 3.566 Å], and in CNO, through C_11_–H_11b_···C_g1_ [*d*(C···C_g_) = 3.727 Å]. This is supported by the HS analysis, which reveals a convex region over aromatic ring 1 and a corresponding concave region over the donor atoms: C_8_–H_8a_ in CNF and C_11_–H_11b_ in CNO (Figure 4c,d). In the *Fingerprint* plots, these C–H···π interactions appear as H···C contacts, accounting for 25.3% in CNF and 23.7% in CNO (Figure 4e,f). Regarding practical applicability, although CNF and CNO are solids at room temperature, it is common in the fuel industry to use solid additives that are previously dissolved in compatible solvents or incorporated into the fuel through specific formulation techniques.

**Figure 4:**
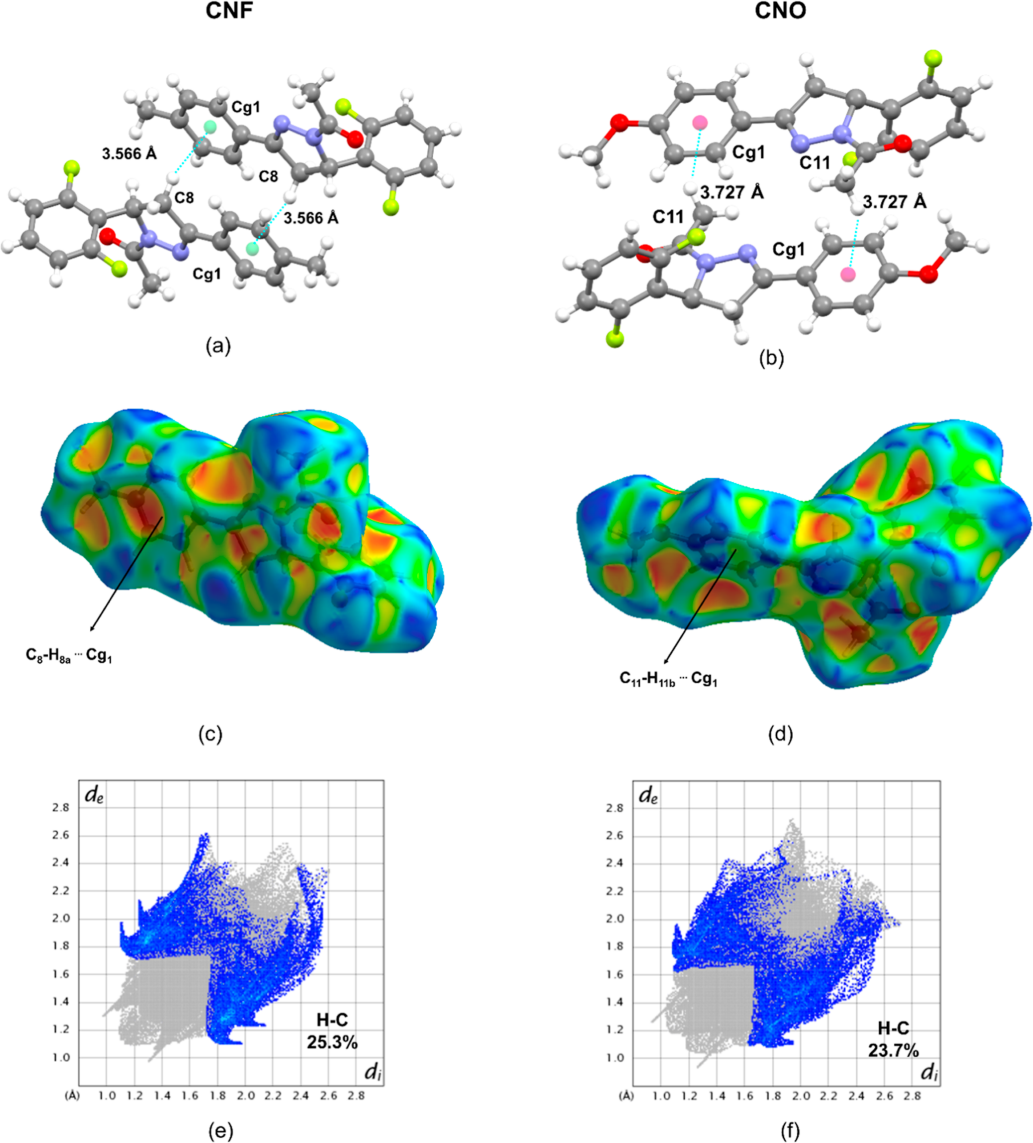
Shape index surface of (a) CNF and (b) CNO. The detailed view of the C–H···π interactions between (c) C_8_–H···C_g1_ for CNF = 3.566 Å and (d) C_11_–H···C_g1_ = 3.727 Å. The 2D fingerprint plot of the C···H for CNF (e) 25.3% and CNO (f) 23.7% of total contacts mapped.

### 3.2 Molecular Modeling Analysis

In both molecules, the HOMO and LUMO are π orbitals; although the HOMO and LUMO orbitals are distributed throughout the molecule, their highest concentration occurs in aromatic rings 2 and 3, showing these regions as the main ones responsible for the electronic interactions of the molecule, both for CNF and CNO. The isosurfaces of these orbitals are depicted in Figure 5. According to the hard–soft acid–base (HSAB) principle,^52^ the energy values of these orbitals suggest that CNF is the most acidic compound. Furthermore, the energy gap values (Δ*E*_H–L_) indicate that the presence of the −OCH_3_ group in the CNO structure makes its molecule slightly more reactive (≈1.04%) compared to CNF, with slightly lower chemical hardness, resulting in greater polarizability (Table 3). The ionization potential and electron affinity show that CNO is the compound that most readily undergoes oxidation during the redox processes.

**Figure 5:**
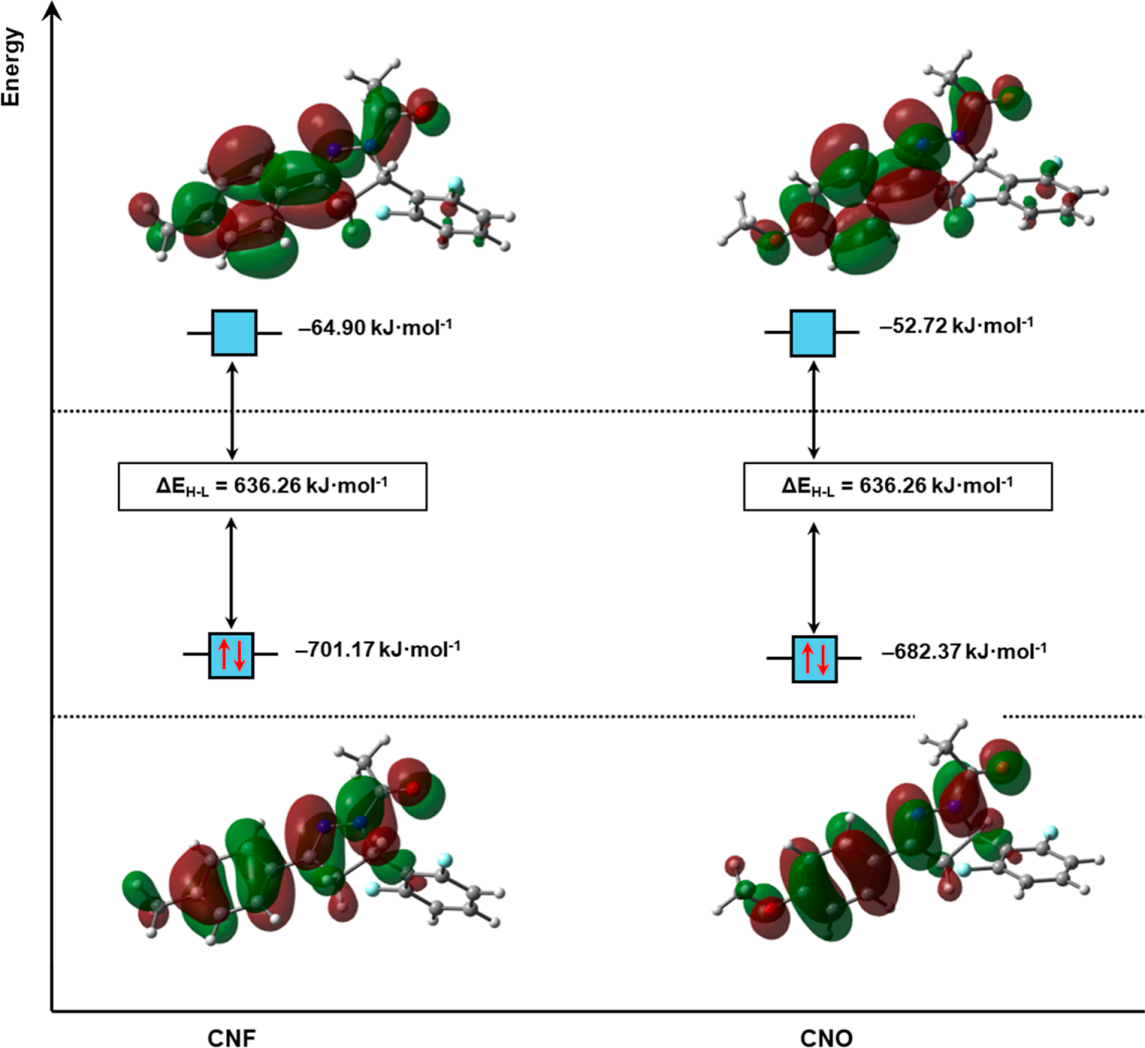
FMO, HOMO (below), and LUMO (above) plots for CNF and CNO calculated at the M06–2X/6–311++G­(d,p) level of theory.

**Table 3:** Chemical Reactivity Descriptors of the CNF and CNO Compounds and Additive Compounds Used in biodiesel^a^ ^,^ ^b^

descriptor	CNF	CNO	BHA	BHT	GA	PG	PY	TBHQ
*E*_HOMO_	–701.17	–682.37	–673.76	–689.04	–763.44	–758.98	–706.97	–685.41
*E*_LUMO_	–64.90	–52.72	–16.51	–17.59	–62.25	–47.60	–29.01	–22.63
Δ*E*_H–L_	636.26	629.65	657.24	671.45	701.19	711.38	677.96	662.78
ionization energy (*I*)	701.17	682.37	673.76	689.04	763.44	758.98	706.97	685.41
electronic affinity (*A*)	64.90	52.72	16.51	17.59	62.25	47.60	29.01	22.63
electronegativity (χ)	383.03	367.54	345.14	353.31	412.85	403.29	367.99	354.02
chemical potential (μ)	–383.03	–367.54	–345.14	–353.31	–412.85	–403.29	–367.99	–354.02
chemical hardness (η)	318.13	314.82	328.62	335.72	350.60	355.69	338.98	331.39
electrophilicity index (ω)	230.59	214.55	181.24	185.91	243.08	228.63	199.74	189.10

a The units are in kJ·mol^–1^.

b Δ*E*_H–L_ = *E*_LUMO_ – *E*_HOMO_.

For commercial compounds used as biodiesel additives, such as BHA, BHT, GA, PG, pyrogallol (PY), and *tert*-butylhydroquinone (TBHQ), the ionization energy ranges from 673 to 764 kJ mol^–1^, while the electron affinity ranges from 16 to 62 kJ mol^–1^ (Figure 6). The corresponding values for CNF and CNO fall within these ranges, suggesting that both possess intrinsic antioxidant potential. Notably, the lower oxidation potential and favorable chemical potential of CNO point to its enhanced ability to transfer charge, a key feature in mitigating oxidative degradation. In general, lower oxidation potentials correlate with a greater tendency to donate electrons and neutralize free radicals, an essential mechanism for preventing peroxidation in biofuels.^53^ Therefore, pyrazoline derivatives with oxidation potentials comparable to or lower than those of benchmark antioxidants such as BHT and TBHQ are expected to effectively delay the onset of oxidation in biodiesel systems, provided they also exhibit adequate miscibility and stability in fuel matrix. Both descriptors for CNF and CNO fall within this range, suggesting that they possess some antioxidant potential. Additionally, the chemical potential of CNO indicates that the compound tends to transfer charge more easily.

**Figure 6:**
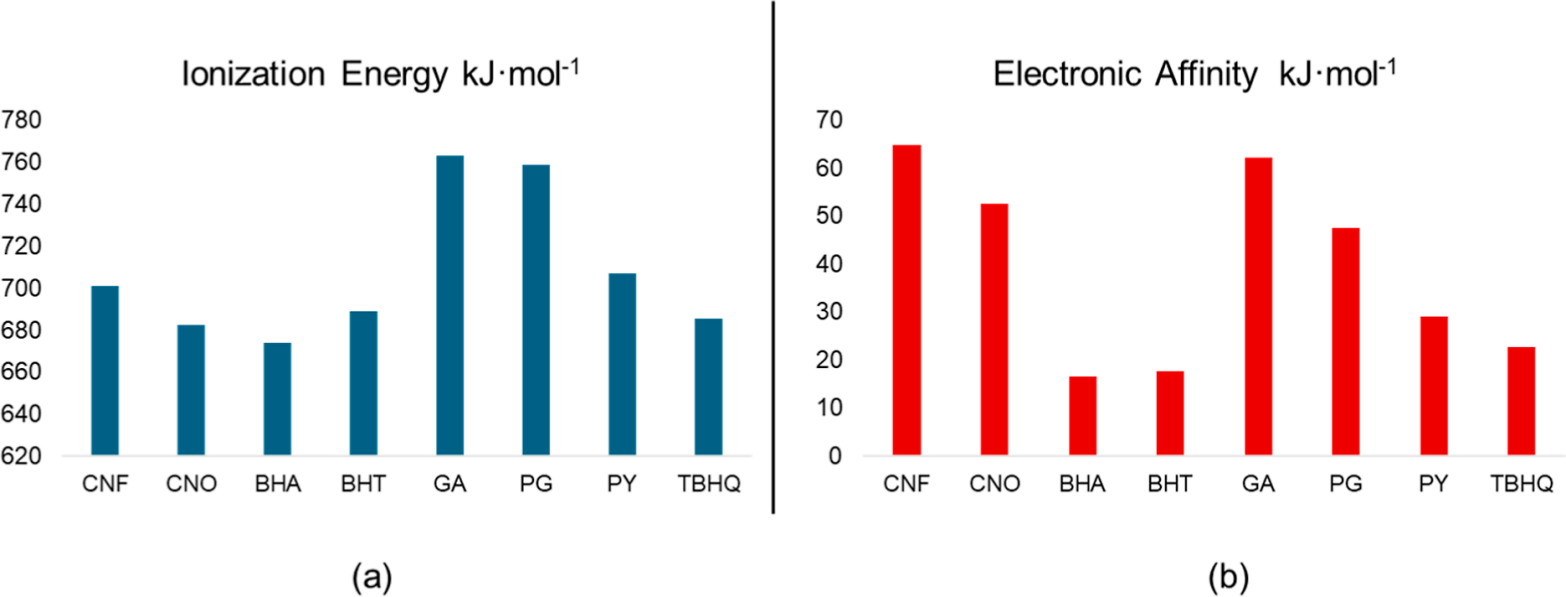
Ionization energy (a) and electronic affinity (b) for CNF and CNO compounds, compared to the commercial additives (CA).

Comparing the values of the global electrophilicity index with the results obtained by Domingo–Pérez,^54,55^ the pyrazoline derivatives CNF and CNO can be considered strong electrophiles (similarly to CA, which are also strong electrophiles). The electrostatic potential maps of the pyrazoline molecules are presented in Figure 7. The analysis of the maps showed that, as expected, the volume of the CNO molecule is approximately 2.68% larger compared to CNF. Another important observation is that the molecular polarity index (MPI)^56^ indicates that the CNO molecule is 6.58% more polar, resulting in a larger polar surface area (52.37%). Compared with the benzene molecule, whose MPI value is 35 kJ·mol^–1^, we can see that both pyrazoline molecules are polar. These differences are due to the presence of the −OCH_3_ group, which is more voluminous than the −CH_3_ group present in ring 3, in addition to containing the ether oxygen atom, which has a high charge density.

**Figure 7:**
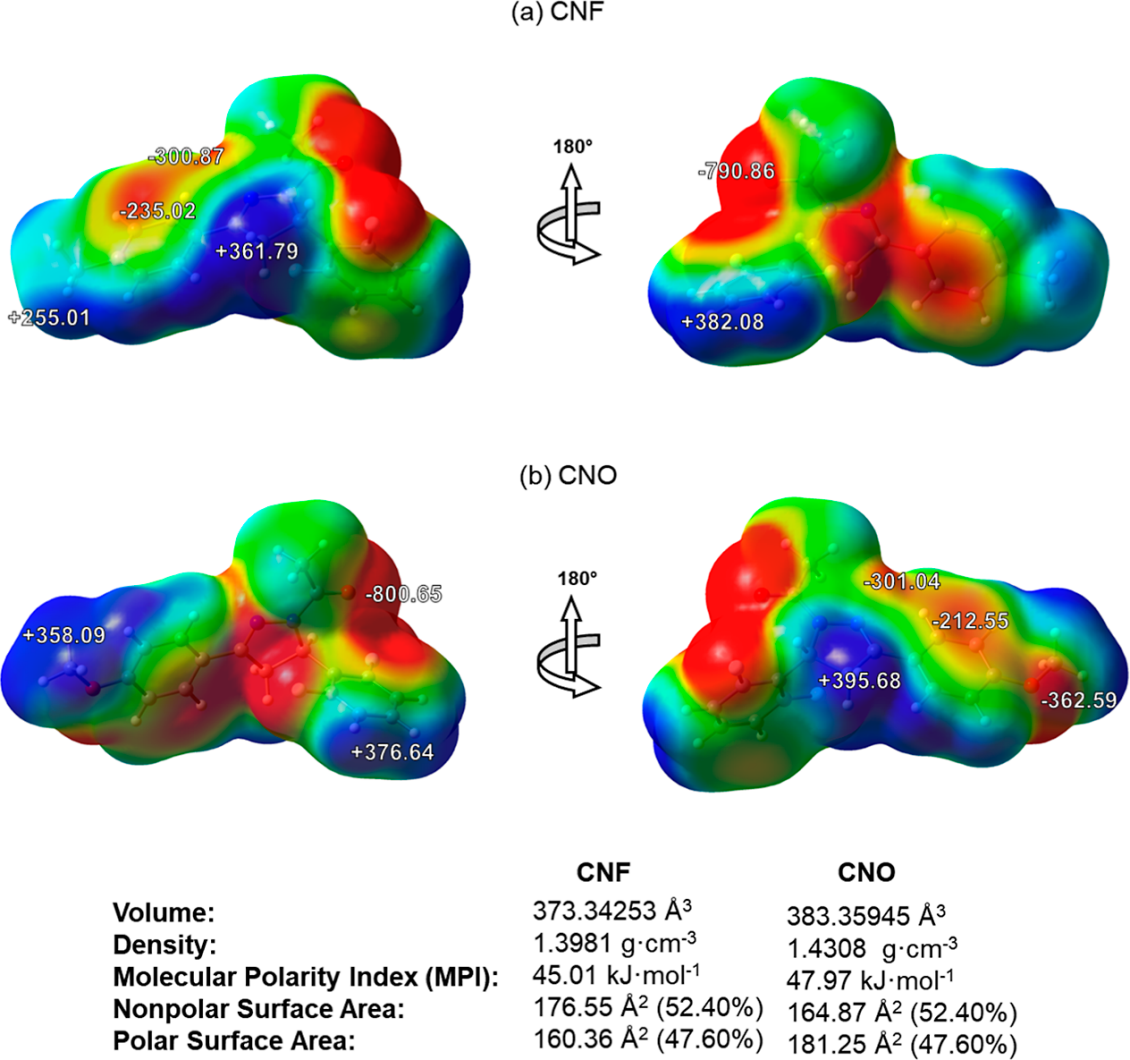
MEP surface at **ρ**(**r**) = 4.0 × 10^–4^ electrons/Bohr^3^ contour of the total SCF electronic density for (a) CNF and (b) CNO, at the M06–2X/6–311++G­(d,p) level of theory. The electrostatic potentials are in kJ·mol^–1^.

The presence of the −OCH_3_ group in CNO increases the *V*(**r**) value in the aromatic portion of ring 3 by 9.56%, which is attributed to the electron-withdrawing effect of this group. However, the carbonyl oxygen atom (O_1_) bonded to the pyrazoline ring becomes 1.24% smaller, making the region over this atom slightly more nucleophilic in CNO. These observations are indicated by the red color on the MEP map, which denotes a high charge density. The −OCH_3_ group also creates a region of high charge density over the O_2_ atom; however, this density is approximately 2.2 times lower than that over O_1_. Another region where the presence of the −OCH_3_ group significantly affects the *V*(**r**) value is around the C_8_ atom in the pyrazoline ring, as indicated by the dark blue color on the map. The electron-withdrawing effect of the substituent group makes this region approximately 9.34% more electrophilic in CNO, leading to an increase in *V*(**r**). Conversely, the regions around the H atoms of C_1_, C_2_, and C_4_ showed a maximum reduction of 1.42% in their charge density.

Beyond these effects, the −OCH_3_ group plays a crucial role in modulating the electronic environment of the molecule. Its electronic effects, including + *I* (inductive) and + *M* (resonance), increase electron density in adjacent aromatic rings, enhancing nucleophilicity and facilitating electron donation during oxidation reactions.^57,58^ This contributes to the stabilization of free radicals, preventing premature fuel degradation. Furthermore, studies of chalcones with methoxy substituents have demonstrated improved antioxidant activity, emphasizing the role of electron-donating groups in oxidation resistance. Additionally, the methoxy group influences the molecular planarity and spatial arrangement, potentially enhancing intermolecular interactions within the fuel matrix. These combined effects contribute to greater oxidative stability, reducing degradation byproducts and extending fuel shelf life.^59^ Beyond its antioxidant activity, the methoxy (−OCH_3_) group has also been reported in the literature for its antibacterial properties, which is advantageous for its application as a biofuel additive.^18,60^

The Fukui function isosurfaces for the CNF and CNO molecules are presented in Figure 8. The *f* ^+^ analyses showed that the C_13_ and C_18_ atoms of ring 3 have the lowest charge density in the molecules and are, therefore, the most susceptible to nucleophilic attacks. In addition to these, the results showed that the C_9_, C_15_, and N_1_ atoms are also susceptible. In the case of the *f* ^–^ function, the analyses indicated that the charge densities on the N_2_ atom are higher, making it the most susceptible to electrophilic attacks. The C_9_ and O_1_ atoms can also experience this type of attack. Finally, the *f* ^0^ analyses indicated that, preferentially, radical attacks can occur on N_2_, followed by N_1_, O_1_, C_9_, and C_15_. The O_2_ atom in CNO can also be attacked by radicals, although with a lower probability compared to the atoms already mentioned.

**Figure 8:**
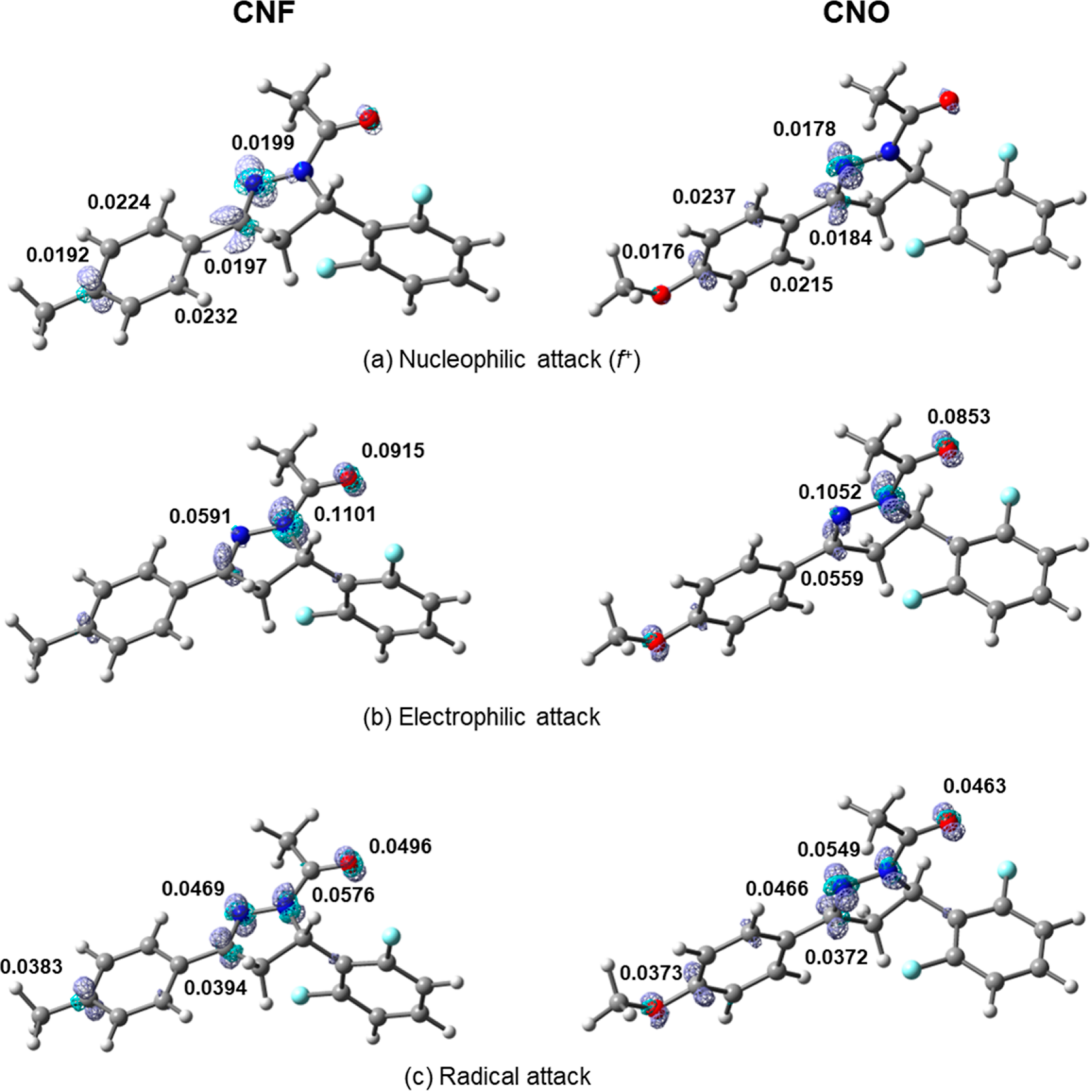
Isosurfaces of the Fukui functions (a) nucleophilic attack ***f*** ^+^, (b) electrophilic attack ***f*** ^–^, and (c) radical attack ***f*** ^0^ for the pyrazoline compounds CNF (left) and CNO (right), obtained at the M06–2X/6–311++G­(d,p) level of theory.

### 3.3 Machine Learning

The reaction rate constant (*k*_OH_) reveals an important kinetic parameter to evaluate the efficiency of the degradation of a compound by the hydroxyl radical attack: a high value indicates faster oxidation. For this discussion, we considered the ML model (XGBoost) and the Morgan fingerprint as structural descriptors with AD % above 18% for these molecules within the training set of this tool, ensuring structural similarity to the compounds in the training set. Table 4 presents the *k*_OH_ values for the major constituents of diesel (represented by the C_10_H_20_ molecule) 1.14 × 10^10^ M^–1^·s^–1^, biodiesel—FAME—represented by methyl 9-octadecenoate (M9OD) 5.94 × 10^9^ M^–1^·s^–1^, MPAL 4.98 × 10^9^ M^–1^·s^–1^ and methyl 8,11-octadecenoate (M8OD) 5.94 × 10^9^ M^–1^·s^–1^; these values serve as reference points for assessing the oxidative potential of antioxidant additives.

We compared the chemical structures in this work to some previous published by our group, focusing on the application as an additive for biodiesel with standardized experiments like the Rancimat EN 15751:2014 ^61^ and heat of combustion ASTM D4809.^62^ Taking the CNF and CNO (this work) as reference molecules, the molecular similarity was evaluated using the Tanimoto index^63^ and mapped over the Morgan fingerprint of 1024 bits^64^ by using the RDKit^65^ and similarity maps^66^ for visualization, against the target compounds Chal01, Chal05,^46^ TMC20,^47^ and the CA;^48^ see Figure S6. The lowest similarity was observed for pyrogallol (PY) (0.082–0.093%), while other compounds exhibited higher similarity values (>21%), with a mean of 0.36–0.37%.

The pyrazoline derivatives showed notable reactivity toward hydroxyl radicals: 1.072 × 10^10^ M^–1^ s^–1^ for CNF (AD = 18.87%) and 1.065 × 10^10^ M^–1^·s^–1^ for CNO (AD = 21.05%) applying the Morgan fingerprint. These values are comparable to or exceed that of TMC20 (9.40 × 10^9^ M^–1^·s^–1^), a trimethoxy chalcone previously reported by our group,^47^ and approach the reactivity potential of Chal01 (4.60 × 10^10^ M^–1^·s^–1^) and Chal05 (1.24 × 10^10^ M^–1^·s^–1^), two highly active arylsulfonamide chalcones.^46^ Notably, all these values surpass the reactivity rates of the reference diesel and biodiesel compounds (BD) (Table 4).

**Table 4:** Reaction Rate (*k*_OH_) for the CNF and CNO (This Work), Chal01 and Chal05,^46^ TMC20,^47^ and Other Commercial Additives (*CA)^48^ ^,^ ^a^

molecule	reaction rate constant *k*_OH_ (M^–1^·s^–1^)	AD (%)
diesel (C_10_H_20_)	6.05 × 10^9^	50
BD M9OD (C_19_H_38_O_2_) 19.98%	5.90 × 10^9^	48.21
BD MPAL (C_17_H_34_O_2_) 12.87%	5.70 × 10^9^	58.7
BD M8OD (C_19_H_34_O_2_) 10.22%	5.90 × 10^9^	40.32
CNF (C_18_H_16_F_2_N_2_O)	1.072 × 10^10^	18.87
CNO (C_18_H_16_F_2_N_2_O_2_)	1.065 × 10^10^	21.05
Chal01 (C_22_H_19_O_4_NS)	4.60 × 10^10^	20.29
Chal05 (C_21_H_16_O_5_N_2_S)	1.24 × 10^10^	24
TMC20 (C_18_H_18_O_4_)	9.40 × 10^9^	33.93
*CA BHT (C_15_H_24_O)	4.16 × 10^9^	100
*CA TBHQ (C_10_H_14_O_2_)	7.57 × 10^9^	100
*CA BHA (C_22_H_32_O_4_)	7.27 × 10^9^	66.67
*CA PG (C_10_H_12_O_5_)	1.11 × 10^10^	100
*CA PY (C_6_H_6_O_3_)	7.06 × 10^9^	40.74
*CA GA (C_7_H_6_O_5_)	4.09 × 10^9^	40.62

a The values were obtained with the ML model XGBoost and the Morgan molecular fingerprints. Diesel and biodiesel (BD)^45^ are represented by their majority compound, respectively. AD is the % of similarity within the applicability domain.

The *k*_OH_ constants were also obtained for compounds used as CA, as previously reported. The results, listed in Table 4, show PG with the highest rate constant (1.11 × 10^10^ M^–1^·s^–1^), followed by TBHQ (7.57 × 10^9^ M^–1^·s^–1^), BHA (7.27 × 10^9^ M^–1^·s^–1^), and PY (4.06 × 10^9^ M^–1^·s^–1^). In contrast, BHT and GA presented the lowest values, 4.16 × 10^9^ M^–1^·s^–1^ and 4.09 × 10^9^ M^–1^·s^–1^, respectively, both lower than biodiesel components. These values suggest that oxidative degradation for the CA PG, TBHQ, BHA and PY, the TMC20, and arylsulfonamide chalcones (Chal01 > Chal05 > PG > CNF ≥ CNO > TMC20 > PY) has a higher oxidation potential than in diesel and biodiesel references, which is consistent with the results obtained experimentally by the accelerated Rancimat method reported by the authors.^46,47^ When compared with the existing additives, such oxidative rates are generally in the same order, which indicates the pyrazoline-derived derivatives could be candidates to retard the degradation of the biodiesel, being suitable sources for the enhancement of oxidative stability. While the antioxidant potential of CNF and CNO is supported by the data presented, we acknowledge that further studies are necessary to fully evaluate the environmental implications of using these molecules as additives, particularly regarding the emission of nitrogen- and fluorine-containing species, which could contribute to environmental pollution or global warming. Nevertheless, the structural and theoretical insights obtained from CNF and CNO can support other studies with this class of molecules or similar ones without these atoms as a comparative parameter. Future studies involving experimental evaluations, such as Rancimat stability tests or engine simulations, are essential to fully confirm its real-world effectiveness, but current computational findings already indicate its potential to mitigate biodiesel oxidation and increase fuel stability.

## 4 Conclusions

The pyrazoline-derived compounds CNF and CNO exhibit promising potential as antioxidant additives for biodiesel applications. Crystallographic analysis revealed that their supramolecular arrangements, stabilized by weak interactions (C–H···F, C–H···O and C–H···π), contribute significantly to molecular stability in the solid state and suggest a favorable capacity for hydrogen donation. These interactions also influence crystal packing, which can modulate electronic properties and enhance the electron-donating ability in antioxidant processes. Chemical reactivity descriptors indicate that CNO, due to the presence of an −OCH_3_ group, has a greater tendency toward oxidation during redox processes, acting as an electron-withdrawing substituent. Although CNF exhibits higher acidity, it is slightly less reactive than CNO in oxidative contexts.

Kinetic evaluations using a machine learning approach (XGBoost with Morgan fingerprints) show that both CNF and CNO possess intermediate oxidation potential when exposed to hydroxyl radicals. Their predicted reaction rate constants are comparable to several CA, trimethoxy and arylsulfonamide chalcones, and fall within the expected range observed in experimental studies using the Rancimat method previously reported by the authors. The oxidative degradation potential follows the trend: Chal01 > Chal05 > PG > CNF ≥ CNO > TMC20 > PY, reinforcing the relevance of these molecules as viable alternatives. Overall, the combination of crystallographic, theoretical, and kinetic data confirms that CNF and CNO are cost-effective candidates for enhancing the oxidative stability of biodiesel. Their balanced performance suggests potential application as next-generation additives in renewable fuel formulations.

## Supplementary Material



## Notes

^1^H NMR and ^13^C NMR spectra of CNF and CNO and crystallographic information file of CNF (Deposition Number: 2393018) and CNO (Deposition Number: 2393138) deposited in the CSD (PDF)
