# Supplementary material for: Comprehensive Analysis of Pyrazoline Analogs: Exploring Their Antioxidant Potential as Biofuel Additives
Source: ACS Omega. 2025 Jul 31;10(36):40843–56. doi: 10.1021/acsomega.5c00398 (PMC12444558; doi:10.1021/acsomega.5c00398)
Supplement: Supplementary file 1 [file ao5c00398_si_001.pdf]

Comprehensive Analysis of Pyrazoline Analogs: Exploring Their Antioxidant Potential as Biofuel Additives

Patricia R.S. Wenceslau [a*], Antônio S.N. Aguiar [b], Vitor S. Duarte [a], Leonardo R. de Almeida [a], Chris H. J. Franco[c], Gilberto L.B. de Aquino[a,d], Jaqueline E. de Queiroz[a], Adriano O. Maldaner[d] and Hamilton B. Napolitano[a*]

[a] Grupo de Química Teórica e Estrutural de Anápolis, Universidade Estadual de Goiás, Anápolis, GO, Brazil.
[b]Laboratório de Novos Materiais, Universidade Evangélica de Goiás, Anápolis, GO, Brazil,
[c] MINDlab: Molecular Design & Innovation Laboratory, Centro de Química Estrutural, Institute of Molecular Sciences, Instituto Superior Técnico, Universidade de Lisboa, Lisboa, Portugal.
[d] Instituto Nacional de Criminalística, Diretoria Técnico Cientifica, Polícia Federal, Brasília, DF, Brasil.

*e-mail: patricia.wenceslaubio@gmail.com; hbnapolitano@gmail.com

Supplementary information

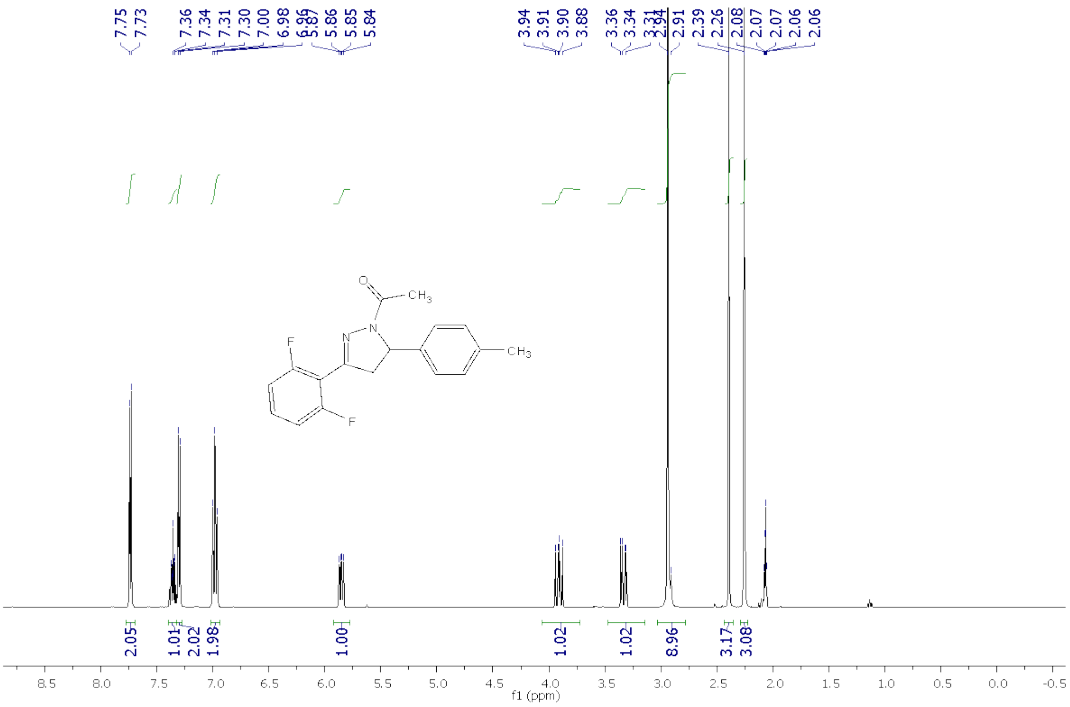

Figure S1. ^{1}H NMR (500 MHz, Acetone-d_6) spectrum for CNF.

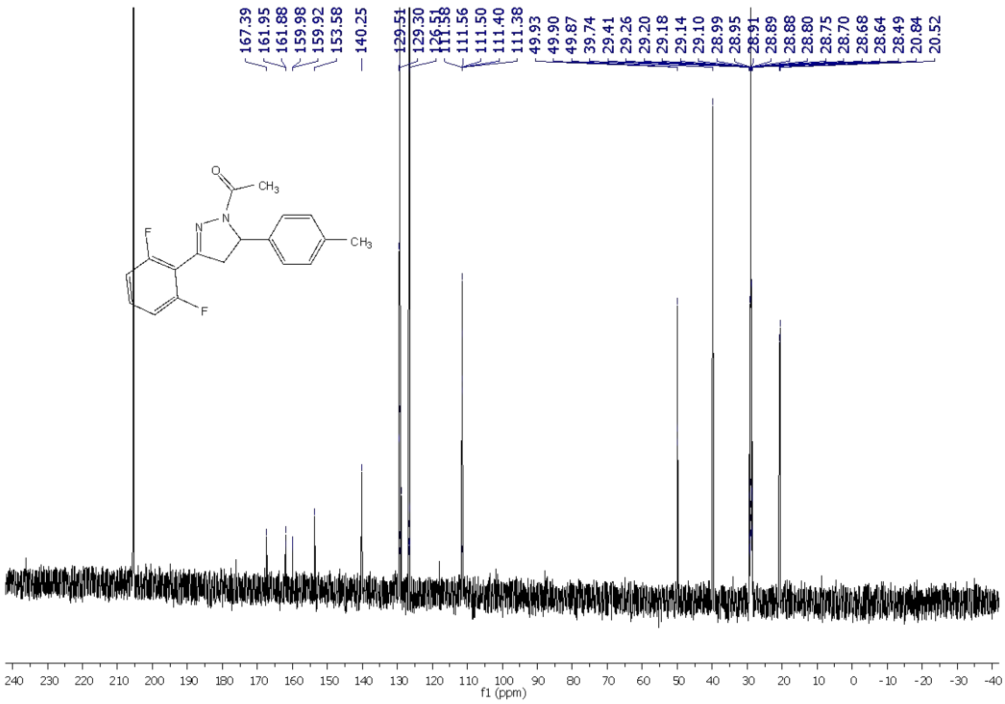

Figure S2. ^{13}C NMR (126, Acetone-d_6) spectrum for CNF.

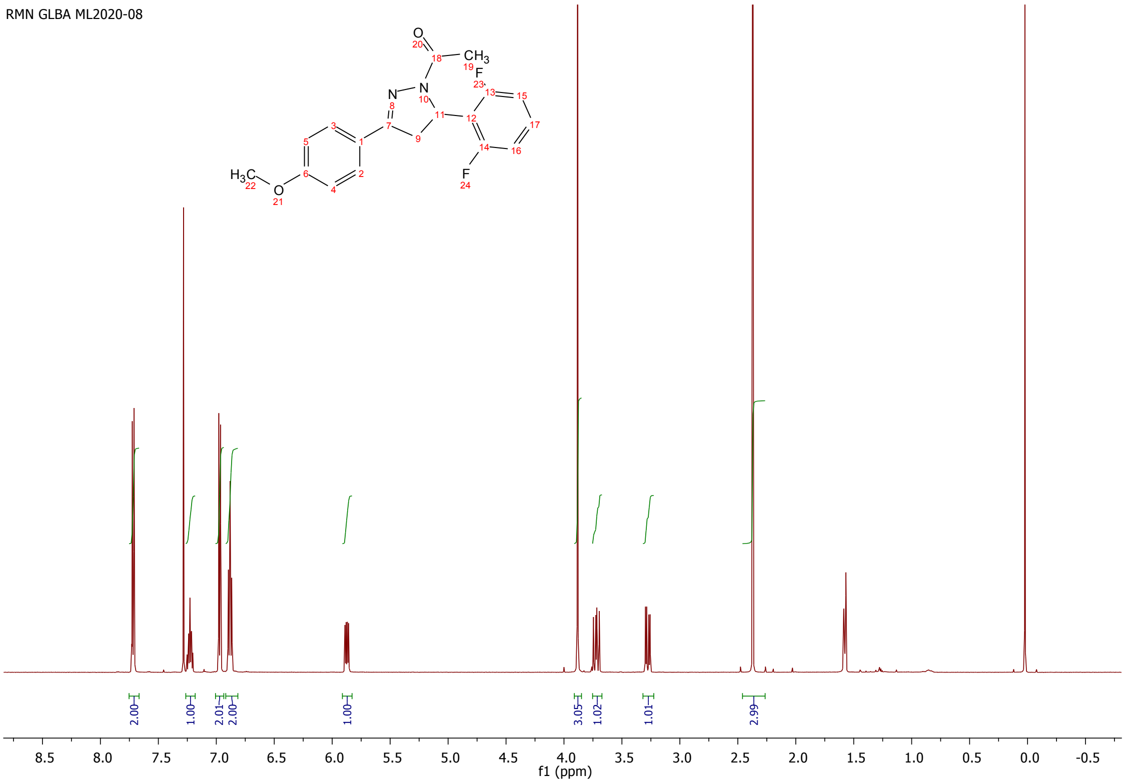

Figure S3. ^{1}H NMR spectrum (500 MHz, Acetone-d6) for CNO.

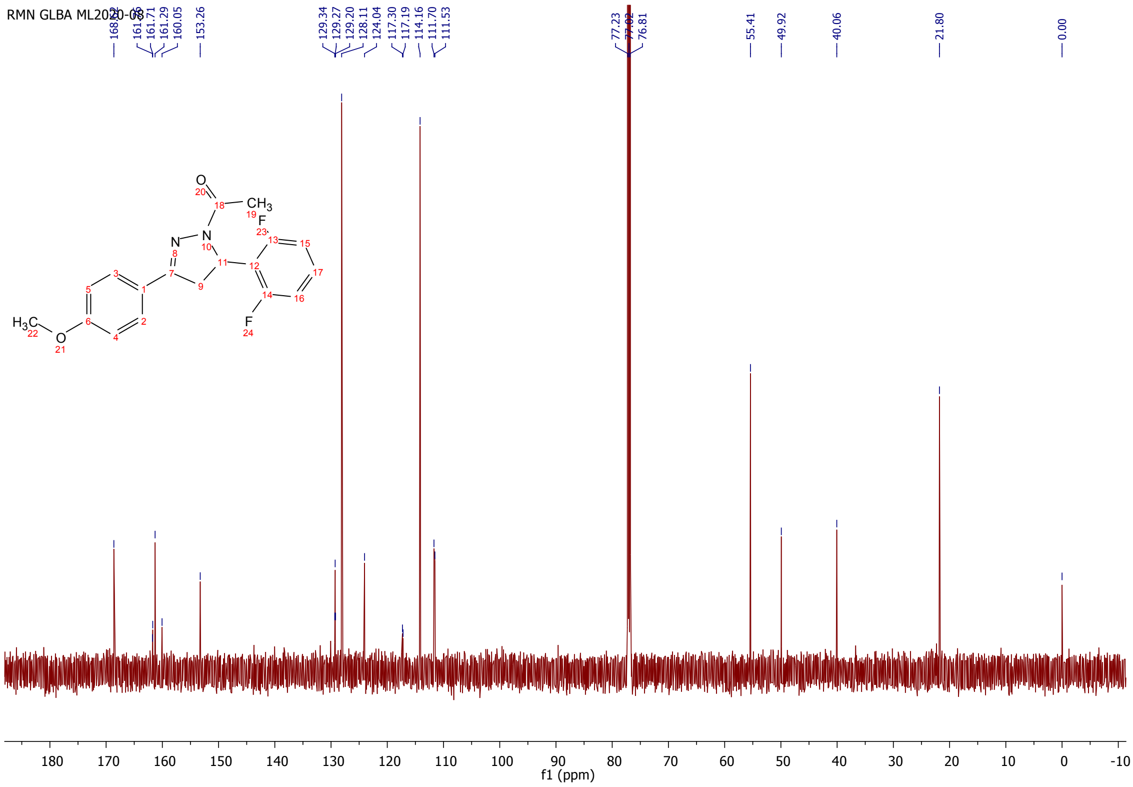

Figure S4. ^{13}C NMR spectrum (126 MHz, Acetone-d6) for CNO.

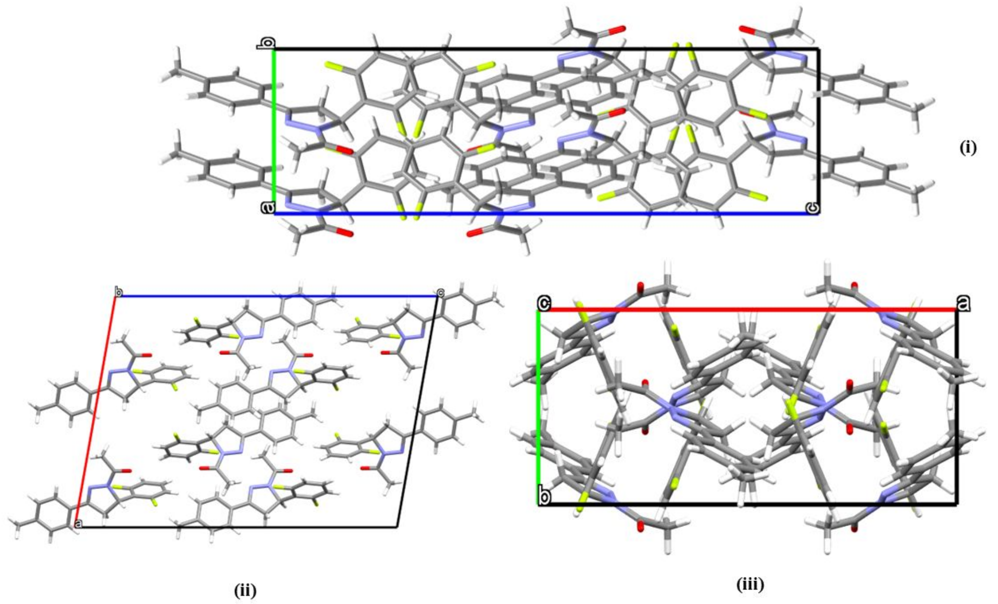

(a)

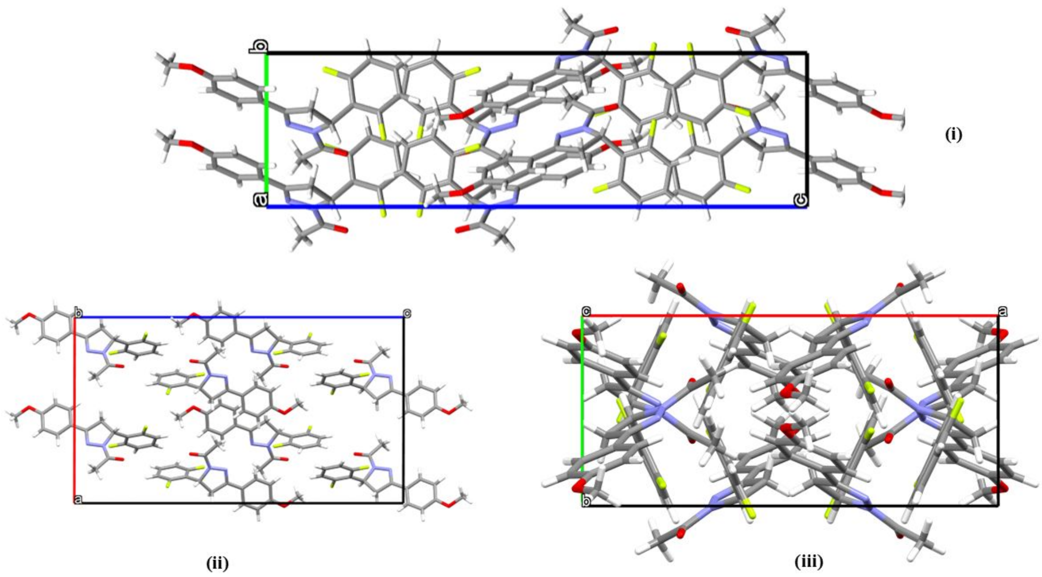

(b)

Figure S5. Molecular packing in the unit cell for CNF (a) and CNO (b). (ai) shows molecular packing along the *a*-axis, (aii) shows molecular packing along the *b*-axis while (aiii) shows molecular packing along the *c*-axis for CNF. For CNO, similarly, representations are shown for *a*-axis (bi), *b*-axis (bii) and *c*-axis (biii).

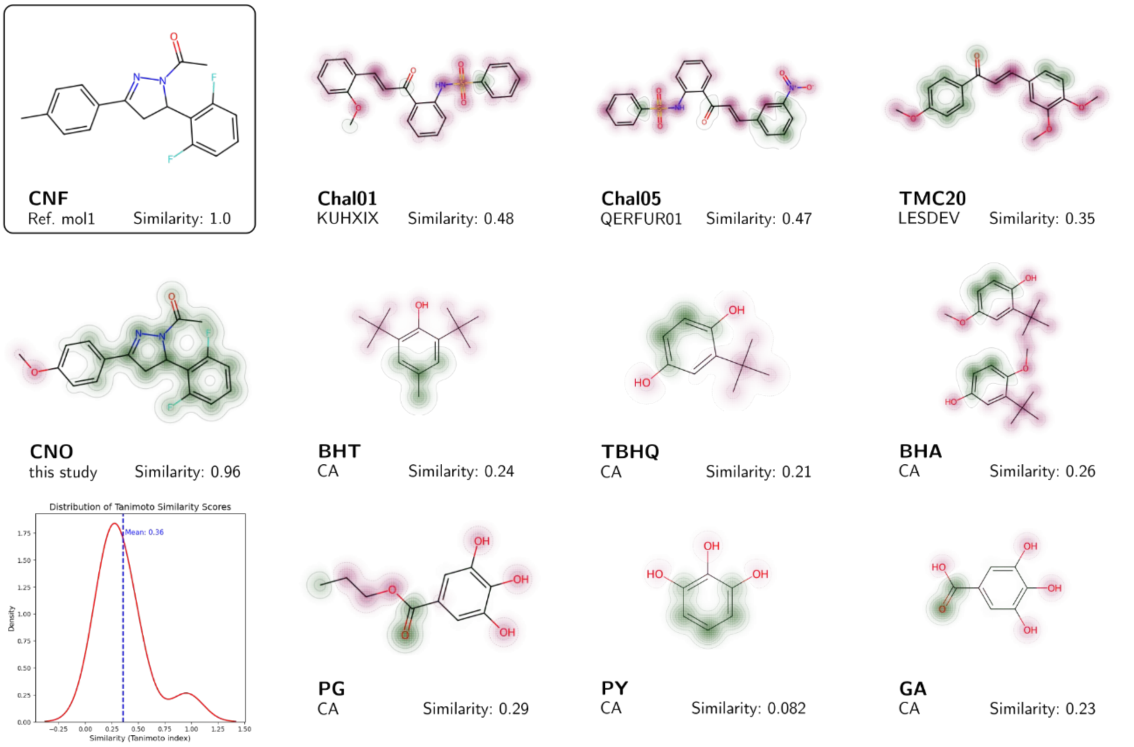

(a)

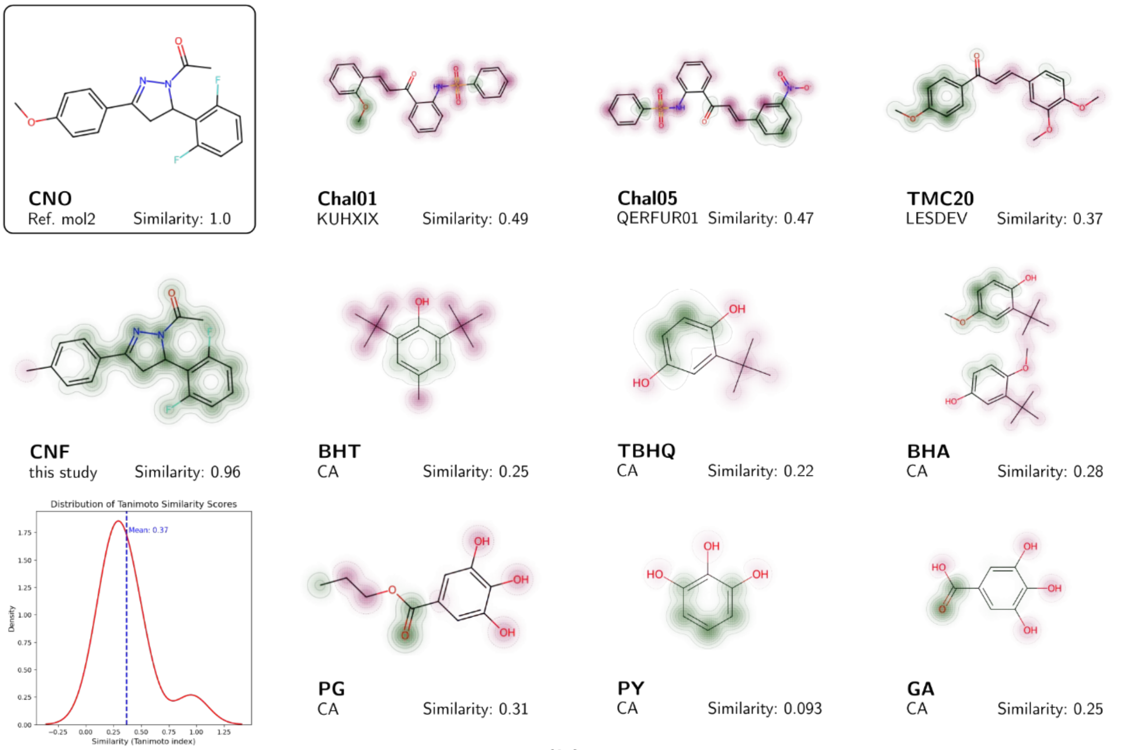

(b)

Figure S6. The maps of molecular similarity obtained by using the *Tanimoto* index compared to (a) CNF and (b) CNO as the reference molecules, and the distribution plot with mean value among them.

Table S1. Fractional Atomic Coordinates (×10^4) and Equivalent Isotropic Displacement Parameters (Å^2×10^3) for CNF at 292.80 (5) K. U_{eq} is defined as 1/3 of the trace of the orthogonalized U_{IJ} tensor.

Atom	x	y	z	U(eq)
O1	7445.2(7)	3756.0(16)	3594.1(5)	69.0(3)
N1	6728.6(6)	5526.6(15)	4739.4(4)	45.6(3)
N2	6812.9(7)	5101.7(15)	4197.5(5)	48.3(3)
C12	5810.8(7)	6842.1(17)	5238.9(5)	40.5(3)
C9	6061.6(7)	6239.6(17)	4723.4(5)	41.1(3)
C6	6361.9(7)	6856.1(18)	3338.6(5)	43.3(3)
C13	5098.9(7)	7648.7(19)	5222.9(5)	45.5(3)
C3	6682.2(7)	8523.8(19)	3469.7(6)	45.1(3)
C7	6137.1(8)	5602.0(18)	3773.0(5)	45.7(3)
C8	5591.3(7)	6385.6(19)	4145.0(5)	44.7(3)
C15	5339.9(8)	8066.1(18)	6226.1(6)	46.4(3)
C18	6283.7(8)	6636(2)	5760.9(6)	50.5(3)
C14	4868.9(8)	8247.7(19)	5709.7(6)	48.7(3)
C17	6050.0(8)	7243(2)	6241.8(6)	52.5(4)
C10	7437.3(9)	4206.5(18)	4077.6(6)	52.2(4)
C5	6295.9(9)	6393(2)	2777.9(6)	55.7(4)
C2	6932.0(9)	9659(2)	3092.2(7)	57.8(4)
C1	6859.9(10)	9092(2)	2543.6(7)	65.0(4)
C16	5093.4(10)	8719(2)	6756.2(6)	62.6(4)
C4	6538.3(10)	7466(3)	2382.2(6)	64.9(4)
C11	8087.5(9)	3845(2)	4547.1(8)	67.4(5)
F2	6757.5(5)	9065.4(11)	4011.7(3)	57.3(3)
F1	5985.0(7)	4778.1(16)	2617.4(4)	85.5(4)

Table S2. Anisotropic displacement parameters (Å^2×10^3) for CNF at 292.80 (5) K. The anisotropic displacement factor exponent takes the form: $-2\pi^2[h^2a^{*2}U_{11}+...+2hka^*b^*U_{12}]$.

**Atom**	**U_{11}**	**U_{22}**	**U_{33}**	**U_{23}**	**U_{13}**	**U_{12}**
O1	87.0(8)	64.5(7)	63.6(7)	-5.3(5)	36.1(6)	14.5(6)
N1	50.6(6)	47.9(6)	41.0(6)	4.3(5)	15.3(5)	7.0(5)
N2	56.1(7)	50.8(6)	41.2(6)	3.0(5)	17.3(5)	11.0(5)
C12	41.7(6)	40.9(6)	40.9(6)	2.0(5)	12.8(5)	-1.7(5)
C9	43.6(6)	40.3(6)	41.4(7)	3.4(5)	13.0(5)	-1.0(5)
C6	42.4(6)	49.8(7)	39.4(6)	1.0(5)	11.6(5)	1.4(5)
C13	44.7(7)	51.9(7)	40.4(6)	4.6(5)	8.4(5)	5.2(6)
C3	40.2(6)	50.9(7)	46.2(7)	0.3(6)	13.0(5)	3.4(5)
C7	50.6(7)	45.5(7)	42.5(7)	-2.9(5)	12.9(5)	-5.3(6)
C8	43.3(7)	51.6(7)	41.1(7)	2.5(5)	12.5(5)	-3.2(5)
C15	51.8(7)	45.0(7)	45.2(7)	-1.4(5)	16.4(6)	-1.6(6)
C18	41.8(7)	63.2(8)	47.1(7)	-1.1(6)	9.4(5)	7.2(6)
C14	46.5(7)	51.6(7)	50.7(7)	3.2(6)	15.6(6)	9.6(6)
C17	49.8(7)	66.6(9)	40.3(7)	-1.4(6)	5.7(5)	3.7(6)
C10	61.7(8)	42.3(7)	59.0(8)	5.2(6)	28.8(7)	6.3(6)
C5	61.3(8)	63.6(9)	43.5(7)	-5.3(6)	12.2(6)	-1.9(7)
C2	52.0(8)	53.8(8)	72.3(10)	10.7(7)	23.6(7)	1.6(6)
C1	65.5(9)	74.6(11)	61.5(9)	22.9(8)	29.1(8)	11.0(8)
C16	69.2(10)	71.0(10)	51.2(8)	-7.8(7)	20.6(7)	5.0(8)
C4	70.5(10)	87.0(12)	40.8(8)	5.9(8)	20.1(7)	10.7(9)
C11	60.0(9)	69.1(10)	78.1(11)	16.3(8)	26.4(8)	20.4(8)
F2	61.8(5)	59.0(5)	52.7(5)	-11.0(4)	14.5(4)	-8.8(4)
F1	117.7(9)	86.6(7)	54.3(6)	-23.9(5)	20.5(5)	-29.3(6)

Table S3. Bond Lengths for CNF at 292.80 (5) K.

**Bonds**	**Length (Å)**	**Bonds**	**Length (Å)**
O1-C10	1.2208(18)	C3-C2	1.374(2)
N1-N2	1.3831(15)	C3-F2	1.3580(15)
N1-C9	1.2848(16)	C7-C8	1.5425(18)
N2-C7	1.4800(18)	C15-C14	1.3827(19)
N2-C10	1.3611(18)	C15-C17	1.388(2)
C12-C9	1.4656(17)	C15-C16	1.5060(19)
C12-C13	1.3859(18)	C18-C17	1.3778(19)
C12-C18	1.3969(18)	C10-C11	1.492(2)
C9-C8	1.5028(17)	C5-C4	1.373(2)
C6-C3	1.3817(19)	C5-F1	1.3540(19)
C6-C7	1.5119(18)	C2-C1	1.379(2)
C6-C5	1.3867(19)	C1-C4	1.369(3)
C13-C14	1.3858(19)		

Table S4. Bond Angles for CNF at 292.80 (5) K.

Bonds	Angle (˚)	Bonds	Angle (˚)
C9-N1-N2	108.33(10)	C8-C7-N2	101.42(10)
C7-N2-N1	113.30(10)	C8-C7-C6	115.87(11)
C10-N2-N1	122.61(12)	C7-C8-C9	102.60(10)
C10-N2-C7	123.96(11)	C17-C15-C14	117.70(12)
C13-C12-C9	120.98(11)	C16-C15-C14	121.52(13)
C18-C12-C9	121.10(11)	C16-C15-C17	120.78(13)
C18-C12-C13	117.92(12)	C17-C18-C12	120.67(12)
C12-C9-N1	120.72(11)	C15-C14-C13	121.32(12)
C8-C9-N1	114.33(11)	C18-C17-C15	121.50(12)
C8-C9-C12	124.96(11)	N2-C10-O1	118.96(14)
C7-C6-C3	123.00(11)	C11-C10-O1	123.10(14)
C5-C6-C3	114.28(12)	C11-C10-N2	117.93(13)
C5-C6-C7	122.65(13)	C4-C5-C6	123.70(15)
C14-C13-C12	120.89(12)	F1-C5-C6	117.66(13)
C2-C3-C6	124.66(13)	F1-C5-C4	118.64(13)
F2-C3-C6	117.27(11)	C1-C2-C3	117.71(15)
F2-C3-C2	118.07(13)	C4-C1-C2	120.80(14)
C6-C7-N2	111.32(10)	C1-C4-C5	118.84(14)
C9-N1-N2	108.33(10)	C8-C7-N2	101.42(10)
C7-N2-N1	113.30(10)	C8-C7-C6	115.87(11)
C10-N2-N1	122.61(12)	C7-C8-C9	102.60(10)
C10-N2-C7	123.96(11)	C17-C15-C14	117.70(12)
C13-C12-C9	120.98(11)	C16-C15-C14	121.52(13)
C18-C12-C9	121.10(11)	C16-C15-C17	120.78(13)
C18-C12-C13	117.92(12)	C17-C18-C12	120.67(12)
C12-C9-N1	120.72(11)	C15-C14-C13	121.32(12)
C8-C9-N1	114.33(11)	C18-C17-C15	121.50(12)
C8-C9-C12	124.96(11)	N2-C10-O1	118.96(14)
C7-C6-C3	123.00(11)	C11-C10-O1	123.10(14)
C5-C6-C3	114.28(12)	C11-C10-N2	117.93(13)
C5-C6-C7	122.65(13)	C4-C5-C6	123.70(15)
C14-C13-C12	120.89(12)	F1-C5-C6	117.66(13)
C2-C3-C6	124.66(13)	F1-C5-C4	118.64(13)
F2-C3-C6	117.27(11)	C1-C2-C3	117.71(15)
F2-C3-C2	118.07(13)	C4-C1-C2	120.80(14)
C6-C7-N2	111.32(10)	C1-C4-C5	118.84(14)

Table S5. Hydrogen Atom Coordinates (Å×10^4) and Isotropic Displacement Parameters (Å^2×10^3) for CNF at 292.80 (5) K.

Atom	x	y	z	U(eq)
H13	4771.3(7)	7789.8(19)	4880.9(5)	54.7(4)
H7	5906.1(8)	4517.9(18)	3587.2(5)	54.8(4)
H8a	5120.0(7)	5693.2(19)	4112.7(5)	53.7(4)
H8b	5463.2(7)	7622.1(19)	4048.0(5)	53.7(4)
H18	6761.2(8)	6084(2)	5784.0(6)	60.6(4)
H14	4388.1(8)	8782.7(19)	5688.7(6)	58.5(4)
H17	6375.2(8)	7099(2)	6584.7(6)	63.0(4)
H2	7142.4(9)	10771(2)	3202.8(7)	69.4(5)
H1	7031.9(10)	9824(2)	2280.2(7)	78.0(5)
H16a	5167(7)	9991(3)	6788(3)	93.9(6)
H16b	4559(2)	8443(15)	6745(2)	93.9(6)
H16c	5397(5)	8140(13)	7073.5(7)	93.9(6)
H4	6484.8(10)	7094(3)	2011.1(6)	77.8(5)
H11a	8093(5)	2598(4)	4643(4)	101.1(7)
H11b	8565.9(12)	4158(18)	4433(2)	101.1(7)
H11c	8022(4)	4548(14)	4867(2)	101.1(7)

Table S6. Fractional Atomic Coordinates (×10[4]) and Equivalent Isotropic Displacement Parameters ($Å^2 \times 10^3$) for CNO at 120 K. U_{eq} is defined as 1/3 of the trace of the orthogonalized U_{IJ} tensor.

Atom	X	y	z	U(eq)
N1	1806.8(6)	5386.5(12)	401.9(3)	16.7(2)
O1	2646.9(5)	3462.6(12)	1462.8(3)	22.3(2)
O2	-162.8(5)	9091.4(13)	-1383.1(3)	24.3(2)
N2	1912.5(6)	4901.2(13)	882.4(3)	17.0(2)
C9	1089.5(6)	6199.6(13)	357.7(3)	15.3(2)
C7	1187.5(6)	5410.5(14)	1192.0(3)	15.8(2)
C12	791.4(6)	6924.1(13)	-104.5(3)	15.5(2)
C6	1424.1(6)	6648.5(14)	1607.5(3)	16.1(2)
C8	587.4(6)	6314.7(14)	820.3(4)	16.2(2)
C13	-23.2(6)	7662.3(14)	-141.4(4)	17.1(2)
C14	-322.0(7)	8369.5(14)	-572.6(4)	18.2(2)
C15	193.1(7)	8348.2(14)	-979.5(4)	17.9(2)
C18	1299.2(6)	6904.1(15)	-516.2(4)	17.7(2)
C10	2569.5(6)	3837.6(14)	1033.8(4)	17.8(2)
C5	1270.4(7)	6156.1(15)	2083.3(4)	19.1(2)
C17	1005.6(7)	7596.9(15)	-953.3(4)	19.2(2)
C2	2010.7(7)	9552.2(15)	1918.5(4)	21.4(2)
C3	1790.6(6)	8377.1(14)	1545.5(4)	17.4(2)
C4	1472.6(7)	7263.8(16)	2473.9(4)	21.6(2)
C1	1852.9(7)	8966.3(17)	2387.4(4)	23.0(2)
C11	3165.1(7)	3156.1(16)	652.3(4)	23.1(2)
C16	348.9(9)	9230(2)	-1804.2(5)	32.3(3)
F2	1942.4(4)	8935.8(9)	1086.3(2)	21.07(19)
F1	907.9(5)	4492.4(10)	2171.0(3)	28.1(2)

Table S7. Anisotropic displacement parameters (Å^2×10^3) for CNO at 120 K. The anisotropic displacement factor exponent takes the form: $-2\pi^2[h^2a^{*2}U_{11}+...+2hka^*b^*U_{12}]$.

Atom	U_{11}	U_{22}	U_{33}	U_{23}	U_{13}	U_{12}
N1	17.7(4)	16.4(4)	16.0(4)	0.2(3)	0.0(3)	0.6(3)
O1	24.2(4)	20.9(4)	21.7(4)	2.7(3)	-2.6(3)	2.7(3)
O2	24.1(4)	29.1(4)	19.7(4)	7.5(3)	-1.8(3)	0.7(3)
N2	16.9(4)	18.1(4)	16.0(4)	0.1(3)	0.8(3)	3.3(3)
C9	16.1(4)	13.4(4)	16.5(4)	-0.5(3)	0.9(3)	-1.3(3)
C7	15.0(4)	15.4(4)	17.0(4)	-0.2(3)	1.5(3)	0.3(3)
C12	15.5(4)	13.4(4)	17.6(4)	0.4(3)	0.2(3)	-1.0(3)
C6	15.4(4)	16.3(4)	16.6(4)	-0.3(3)	0.5(3)	1.0(3)
C8	14.9(4)	16.4(4)	17.2(4)	0.0(3)	0.7(3)	0.4(3)
C13	17.2(4)	15.9(4)	18.1(4)	-0.3(3)	1.3(3)	0.6(3)
C14	17.4(5)	16.4(4)	20.9(5)	0.5(3)	-0.7(3)	1.3(4)
C15	19.2(5)	15.7(4)	18.7(5)	1.9(3)	-2.0(3)	-2.7(4)
C18	15.3(4)	18.1(4)	19.8(5)	1.2(3)	1.6(3)	-0.5(3)
C10	16.9(5)	14.4(4)	22.0(5)	-0.3(3)	-1.2(3)	0.8(3)
C5	20.4(5)	18.0(5)	18.9(5)	1.4(3)	1.6(3)	-0.7(4)
C17	18.4(5)	20.4(5)	18.9(5)	2.2(3)	2.4(3)	-2.1(4)
C2	21.5(5)	20.1(5)	22.5(5)	-2.9(4)	-0.6(4)	-3.1(4)
C3	17.4(4)	18.8(5)	16.1(4)	0.7(3)	1.0(3)	-0.6(4)
C4	23.3(5)	25.3(5)	16.3(5)	-0.6(4)	0.5(3)	2.6(4)
C1	24.0(5)	25.5(5)	19.6(5)	-4.6(4)	-1.9(4)	0.2(4)
C11	20.6(5)	22.9(5)	25.9(5)	-1.0(4)	1.9(4)	7.4(4)
C16	29.9(6)	46.1(8)	21.0(5)	10.2(5)	0.0(4)	-3.5(6)
F2	24.0(3)	21.3(3)	17.9(3)	2.1(2)	3.3(2)	-4.4(2)
F1	39.0(4)	22.2(4)	23.1(4)	4.2(2)	4.7(3)	-8.2(3)

Table S8. Bond Lengths for CNO at 120 K.

Bonds	Length (Å)	Bonds	Length (Å)
N1-N2	1.3889(12)	C6-C5	1.3901(14)
N1-C9	1.2864(14)	C6-C3	1.3883(16)
O1-C10	1.2271(13)	C13-C14	1.3842(14)
O2-C15	1.3638(13)	C14-C15	1.3958(15)
O2-C16	1.4280(15)	C15-C17	1.4005(16)
N2-C7	1.4841(14)	C18-C17	1.3918(14)
N2-C10	1.3608(14)	C10-C11	1.5039(15)
C9-C12	1.4633(14)	C5-C4	1.3846(15)
C9-C8	1.5151(14)	C5-F1	1.3547(14)
C7-C6	1.5062(14)	C2-C3	1.3832(15)
C7-C8	1.5471(14)	C2-C1	1.3919(16)
C12-C13	1.4018(15)	C3-F2	1.3592(12)
C12-C18	1.3999(14)	C4-C1	1.3911(18)

Table S9. Bond Angles for CNO at 120 K.

Bonds	Angle (°)	Bonds	Angle (°)
C9-N1-N2	108.16(8)	C13-C14-C15	119.71(10)
C15-O2-C16	117.71(10)	O2-C15-C14	114.74(10)
N1-N2-C7	113.68(8)	O2-C15-C17	125.07(10)
C10-N2-N1	122.00(9)	C14-C15-C17	120.19(9)
C10-N2-C7	123.73(9)	C17-C18-C12	121.06(10)
N1-C9-C12	122.07(9)	O1-C10-N2	119.96(10)
N1-C9-C8	114.28(9)	O1-C10-C11	123.33(10)
C12-C9-C8	123.64(9)	N2-C10-C11	116.70(10)
N2-C7-C6	113.49(8)	C4-C5-C6	123.73(10)
N2-C7-C8	101.25(8)	F1-C5-C6	118.24(9)
C6-C7-C8	114.33(9)	F1-C5-C4	118.02(10)
C13-C12-C9	119.76(9)	C18-C17-C15	119.40(9)
C18-C12-C9	121.78(9)	C3-C2-C1	117.89(11)
C18-C12-C13	118.46(9)	C2-C3-C6	124.36(10)
C5-C6-C7	122.12(10)	F2-C3-C6	117.29(9)
C3-C6-C7	122.88(9)	F2-C3-C2	118.35(10)
C3-C6-C5	114.99(9)	C5-C4-C1	118.40(10)
C9-C8-C7	102.60(8)	C4-C1-C2	120.62(10)
C14-C13-C12	121.17(9)		

Table S10. Hydrogen Atom Coordinates (Å×10^4) and Isotropic Displacement Parameters (Å^2×10^3) for CNO at 120 K.

Atom	x	y	z	U(eq)
H11A	3652.56	2618.69	802.58	35
H11B	3335.13	4174.55	452.79	35
H11C	2887.65	2239.93	458	35
H16A	47.3	9890.74	-2050.06	48
H16B	483.98	8010.71	-1918.12	48
H16C	859.36	9880.14	-1727.34	48
H4	1379(12)	6860(30)	2799(6)	27(4)
H1	1986(11)	9770(30)	2651(6)	28(4)
H8A	48(10)	5670(20)	799(6)	20(4)
H13	-380(10)	7670(20)	135(5)	17(3)
H7	968(11)	4250(20)	1329(6)	24(4)
H2	2251(12)	10750(30)	1831(6)	29(4)
H17	1385(12)	7590(30)	-1227(6)	31(4)
H8B	446(10)	7590(20)	908(6)	23(4)
H14	-893(12)	8850(30)	-605(7)	32(5)
H18	1863(12)	6420(30)	-493(7)	33(5)
